# The DONE framework: Creation, evaluation, and updating of an interdisciplinary, dynamic framework 2.0 of determinants of nutrition and eating

**DOI:** 10.1371/journal.pone.0171077

**Published:** 2017-02-02

**Authors:** F. Marijn Stok, Stefan Hoffmann, Dorothee Volkert, Heiner Boeing, Regina Ensenauer, Marta Stelmach-Mardas, Eva Kiesswetter, Alisa Weber, Harald Rohm, Nanna Lien, Johannes Brug, Michelle Holdsworth, Britta Renner

**Affiliations:** 1 Department of Psychological Assessment & Health Psychology, University of Konstanz, Konstanz, Germany; 2 Institute of Business Administration, esp. Marketing, Kiel University, Kiel, Germany; 3 Institute for Biomedicine of Aging, Friedrich-Alexander-Universität Erlangen-Nürnberg, Erlangen, Germany; 4 Department of Epidemiology, German Institute of Human Nutrition, Potsdam-Rehbruecke, Germany; 5 Experimental Pediatrics and Metabolism, University Children’s Hospital, Heinrich Heine University Düsseldorf, Düsseldorf, Germany; 6 Department of Pediatric Gastroenterology and Metabolic Diseases, Poznan University of Medical Sciences, Poznan, Poland; 7 Institute of Social Pediatrics and Youth Medicine, Ludwig-Maximilians-University of Munich, Munich, Germany; 8 Chair of Food Engineering, Technische Universität Dresden, Dresden, Germany; 9 Faculty of Medicine, University of Oslo, Oslo, Norway; 10 EMGO Institute for Health & Care Research and the Department of Epidemiology & Biostatistics, VU University Medical Center, Amsterdam, the Netherlands; 11 School of Health and Related Research, University of Sheffield, Sheffield, United Kingdom; Duke University, UNITED STATES

## Abstract

The question of which factors drive human eating and nutrition is a key issue in many branches of science. We describe the creation, evaluation, and updating of an interdisciplinary, interactive, and evolving “framework 2.0” of Determinants Of Nutrition and Eating (DONE). The DONE framework was created by an interdisciplinary workgroup in a multiphase, multimethod process. Modifiability, relationship strength, and population-level effect of the determinants were rated to identify areas of priority for research and interventions. External experts positively evaluated the usefulness, comprehensiveness, and quality of the DONE framework. An approach to continue updating the framework with the help of experts was piloted. The DONE framework can be freely accessed (http://uni-konstanz.de/DONE) and used in a highly flexible manner: determinants can be sorted, filtered and visualized for both very specific research questions as well as more general queries. The dynamic nature of the framework allows it to evolve as experts can continually add new determinants and ratings. We anticipate this framework will be useful for research prioritization and intervention development.

## Introduction

Human food choice, eating behavior, and nutrition form a fascinating and complex behavioral system. While parts of the behavioral repertoire are innate, many learned and modifiable factors also shape nutrition and eating, making the system highly adaptive. This becomes obvious when we consider that food choices and eating behaviors show great diversity across individuals, and populations. Food choices and eating behaviors represent a core aspect of our everyday life with people making more than 200 food decisions daily [1] (see also Hofmann et al. [2] for ecological assessment of eating events, and see Baranowski et al. [3] for a theoretical discussion). Hence, nutrition and eating are likely to be driven by the interplay of numerous factors from the individual to the environmental level. In the current article, we describe the creation, evaluation, and updating of the DONE framework, an interdisciplinary framework of the factors that shape nutrition and eating. The dynamic and interactive character of the DONE framework sets it apart from earlier, more static, frameworks, and it is a pioneering example of a framework 2.0: a new generation of frameworks that continue to evolve and be updated after their release and which may drive and inform further research, practice, and policy making.

### About determinants of nutrition and eating

Different disciplines have developed elaborate and useful frameworks and models for systematizing the correlates and determinants of nutrition and eating. The range of personal, social, economic, and environmental factors influencing these outcomes discussed within the different disciplines is impressive. However, in most cases, the frameworks and models show the signature of the respective discipline. For example, in consumer research the core focus is often on food choice and intrinsic and extrinsic product characteristics such as appearance, packaging, and price as determinants [4] (for a review of the types of determinants considered most frequently in consumer research, see Symmank et al [5]). In contrast, in psychology the focus is mainly on eating behavior and individual trait and state variables such as attitudes, motives, knowledge, and self-control, which are used to predict eating behavior and food choice. Diverse psychological models such as the theory of planned behavior [6], social cognitive theory [7], and the protection motivation [8] theory all assume that behavior is shaped by an expectancy-value calculus, i.e., people strive to optimize positive outcomes and minimize negative outcomes in relation to their respective capacity and ability [3,9,10]. Also stage theories such as the Transtheoretical Model [11], the Precaution Adoption Process Model [12], and the Health Action Process Approach [9,13] assume that the core determinants are individual beliefs and capacities although their relative weight might change across the course of behavior. In the fields of public health and nutritional sciences, biological and psychological determinants are often combined with environmental determinants such as availability and accessibility of food options, economic constraints, and political regulations, laws, and rules. For example, the framework for weight gain prevention (EnRG) assumes that both psychological as well as environmental variables determine dietary behavior [14,15] (see also the food choice process model [16] for another example).

These more discipline-oriented models contribute to our in-depth understanding of certain fields and certain types of determinants such as product characteristics or individual beliefs and attitudes. However, a consequence of the in-depth analysis of certain types of determinants is often that other aspects are comparably neglected. Hence, cross-talk between disciplines is by definition limited due to differences in focus and terminology, and a comparison and integration of the multitude of empirical studies is difficult to achieve (see also Hummel & Hoffmann [17], Sleddens et al. [15], and Symmank et al. [5]).

A need to integrate the correlates and determinants of nutrition and eating across fields has been voiced by different researchers and has led to the conceptualization of integrative frameworks. For example, Booth et al. [18] created a socio-ecological framework encompassing environmental and societal factors affecting food choice and physical activity, which includes eight different layers (psychobiological core, cultural, societal, enablers of choice, lifestyle, behavior settings, proximal leverage points, and distal leverage points) and 65 different correlates and determinants (see also Contento [19]). In a similar vein, Glass and McAtee [20] suggested a multidimensional framework including eight nested hierarchies of different levels of determinants ranging from the global level (including geopolitical and economic factors), via the environmental level, to the individual genomic substrate level. Recently, Bock et al. [21] suggested a two-dimensional food consumption map including four main categories (primary appetite control, dietary choices, food access / affordability, and food supply /offer) which are divided into 12 different clusters (e.g., environment, economy, physiology, individual factors) including more than 150 determinants (e.g., number of fast food restaurants, perception of healthiness) based on consulting with experts from different fields.

The different integrative frameworks show the great complexity of the factors shaping nutrition and eating and provide greater capacity for integrating the multitude of empirical studies than models emerging from one single discipline. However, it appears unlikely that one definitive framework can be established since new developments constantly expand the scope and focus of such frameworks. As a consequence, it appears inherent that frameworks are potentially quickly outdated, if they include specific determinants that can be empirically measured (e.g., number of fast food restaurants), or the categories are too generic (e.g., primary appetite control, societal level, behavior settings) to guide actual research. Hence, although these static frameworks more or less provide a snapshot of the current state of the art, a dynamic approach that allows for continued evolution, including extensions in both the breadth and depth of an integrative framework according to ongoing developments in research, is required. While a need for such integrative frameworks has long been recognized, advances in technology play a large role in making such a dynamic approach possible. These advances allow for comprehensive visualization of complex frameworks, facilitate easy communication and data sharing across countries and disciplines, and make ongoing discussion and updating of frameworks possible.

### Building a framework 2.0 for determinants of nutrition and eating: The DONE framework

**Fig 1:**
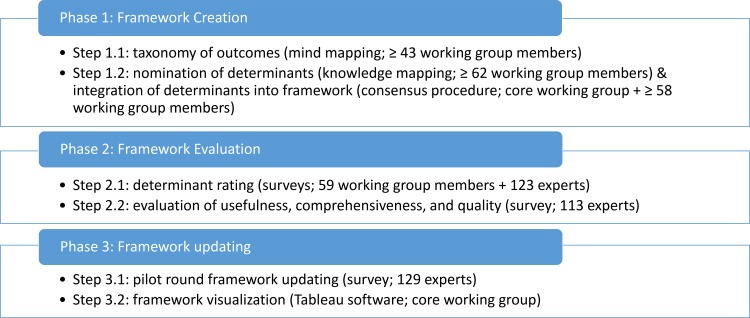
Graphical representation of the methodological approach to creating, evaluating, and updating the DONE framework. The numbers of workgroup members provided involved in each phase are minimum numbers. Actual numbers are likely to be higher since, in some cases, several workgroup members submitted one joint response.

We describe the development of a dynamic, interdisciplinary framework 2.0 of the determinants of nutrition and eating (DONE). The DONE framework encompasses determinants related to nutrition and eating as discussed in different disciplines and is visualized dynamically, making use of new advances in technology, allowing for an interactive, user-friendly representation of the DONE framework. The development of the integrative framework is an ongoing, dynamic process, the starting point of which is outlined in this article. We provide a detailed description of the multiphase, multi-method approach (see Fig 1) employed for the creation, evaluation, and updating of the DONE framework, and present empirical data and results for each of these three phases. This work was undertaken in the context of the European research network and knowledge hub DEDIPAC (Determinants of Diet and Physical Activity [22]). This project involved, among other activities, the development of a framework of the factors shaping nutrition and eating across the lifespan, with the aim of assembling available scientific knowledge on the topic across disciplines in one interdisciplinary framework. This task was undertaken by a workgroup of scholars with varying academic backgrounds and from different countries (see Table 1 for more details). A total of 87 workgroup members contributed to at least one step of the framework creation and evaluation process. The workgroup was managed and guided by a core workgroup led by the first two authors (MS and BR); the other members of the core workgroup (SH, DV, HB, RE, MSM, EK, AW, and MH) guided one of five subgroups. Not all workgroup members contributed to each step in the process of developing and evaluating the framework; Fig 1 indicates the number of workgroup members involved in each step. The workgroup followed earlier successful examples of multidisciplinary partnerships aiming to propel social changes [23] and comprehensively describe the factors influencing obesity-related behaviors [18].

**Table 1:** Scientific backgrounds and countries represented within the workgroup members and external experts participating in the creation, evaluation and updating of the DONE framework.

	Workgroup members (*N* = 87)	External experts (*N* = 129)
**Division of age group expertise**	• 15% children • 65% adults • 20% elderly	• 30% children • 57% adults • 13% elderly
**Scientific backgrounds**	• Anthropology • Biology / Human Biology • Dietetics • Economics • Epidemiology • Food Engineering • Food Science • Food Technology • Geriatrics • Health Promotion • Marketing and Consumer Research • Medicine • Nutrition Science • Pediatrics • Physical Education • Physiology • Physiotherapy • Psychiatry • Psychology • Public Health • Social Demography • Sports Sciences • Statistics	• Biology / Human Biology • Biometry • Economics / Health Economics • Educational Sciences • Environmental Science • Epidemiology • Food and Nutrition Science • Genomics • Geography • Human Ecology • Human-Computer Interaction • Marketing • Mathematics • Medicine • Nursing Science • Policy • Psychiatry • Psychology • Public Health • Sociology • Sports and Physical Activity Science • Statistics
**Countries**	• Belgium • Finland • France • Germany • Ireland • Italy • Netherlands • Norway • Poland • Spain • United Kingdom	• Germany • Italy • Belgium • Netherlands • France • Ireland • Finland • UK • Denmark • Austria • Poland • Switzerland • Marocco • United States

Note: not all workgroup members and external experts participated in every phase; specific numbers are provided for each phase in the methods sections. Fifty-seven of the 129 external experts chose to remain anonymous; their exact academic backgrounds and countries are unknown and thus not included in this table. However, as only scholars with relevant expertise were invited to participate, it can be assumed that all anonymous experts had the necessary expertise to contribute to the development of the framework.

## Phase 1: Framework creation

The main aim of the creation process was to generate a comprehensive and interdisciplinary framework of the determinants of nutrition and eating by using a socio-ecological approach and taking differences between age groups into account. Free nomination of determinants, followed by a multiple-round discussion and consensus approach, was employed to create an interdisciplinary set of determinants and correlates for nutrition and eating and order these factors into a hierarchical, systematic structure. The creation of the framework consisted of two main steps; a graphical representation of the method is depicted in Fig 1.

### Method

#### Step 1.1

Before nominating determinants and correlates, workgroup members first participated in an online mind mapping [24] procedure to identify the outcomes (diet, eating behavior, nutrition, food choice, etc.) for which we aimed to collect influencing factors. This step was deemed necessary to ensure common understanding and a shared language across the members included in the interdisciplinary workgroup. MindMeister software (www.mindmeister.com) was used to facilitate the mind mapping procedure. An online mind map was created, and workgroup members were invited to contribute all outcomes they employed themselves as well as outcomes they encountered in their research to this mind map. They were instructed not to delete any information, but only to add outcomes. Workgroup members could also indicate relations between the various outcomes and comment on their own and others’ actions. The resulting mind map was reduced and structured into a systematic taxonomy by the lead authors (FMS and BR). This taxonomy was subsequently discussed with the workgroup members in a live meeting, which led to further alterations and reductions of the taxonomy and which resulted in a final version of the taxonomy. For more methodological details regarding this step, please refer to Stok and colleagues (manuscript submitted for publication).

#### Step 1.2

Step 1.2 took place in five subgroups of DEDIPAC. Four subgroups covered the core age groups across the lifespan (one children subgroup, two adult subgroups, and one elderly subgroup), and one subgroup focused specifically on nutrition and eating in ethnic minority populations. Each subgroup had one appointed subgroup leader. In each of the five groups, knowledge mapping [25] was used to allow the workgroup members to systematically nominate all the influential factors they considered relevant for one or more of the outcomes identified in Step 1.1. It was explicitly mentioned that the nomination of factors was possible both based on scientific evidence (e.g. literature review; bottom-up nomination) and their expert knowledge and judgment (top-down nomination) in order to generate the most comprehensive and complete framework possible. Concurrent with recent theorizing [16,21] about the importance of distinguishing between different spheres of socio-ecological influence when describing factors influencing health behavior (including nutrition and eating), we aimed for the framework to broadly follow a socio-ecological approach and therefore asked workgroup members to sort each nominated factor into a predefined systematic structure comprised of four main socio-ecological levels: Individual, Interpersonal, Environmental, and Policy. Workgroup members contributed their determinants via e-mail to their respective subgroup leader, in the form of a list of determinants in Microsoft Excel® and/or a model of determinants depicted in a Microsoft Powerpoint® slide.

Subsequently, the core workgroup, comprising representatives of each subgroup, completed three structured discussion rounds (one of which occurred via e-mail and video conference, and two of which were live discussion sessions). For each discussion round, the first author prepared a tentative framework, indicating the changes made from the previous version(s), and a list of points for discussion. All changes, unclarities and disagreements were extensively discussed until agreement was reached among the core workgroup members. Three discussion rounds were held because the number of determinants (and, as a consequence, the number of issues to discuss) was large, and core workgroup members’ time was limited at each opportunity for discussion. The aim of these discussion rounds was to integrate and synthesize the determinants into one overarching life course framework with a clear structure, to remove duplicate factors, and to streamline factor names. Within the four main socio-ecological levels, two additional sorting layers (stem-categories and leaf-categories) were discussed and agreed upon, giving the framework a fine-grained three-layer structure. The framework is constructed so that it comprises two main age groups, *children* and *adults*, each with a separate and complete set of influencing factors specified in the framework. In addition, the framework includes factors unique to specific subgroups of these two age groups: *infants* and *school-aged children* are subgroups of the *children* age group, while *elderly* is a subgroup of the *adults* age group. The division into age groups was decided upon because not all factors may influence all age groups and, most importantly, because the relevance of any given factor may differ between age groups. A number of determinants identified by the ethnic minority populations subgroup were deemed exclusively influential for nutrition and eating in ethnic minority populations. These determinants are denoted in the framework by the prefix EM (Ethnic Minority).

In a final consensus round, all members of the workgroup gave feedback on the synthesized framework to ensure that it represented the expert concepts they had originally contributed. This consensus round occurred via e-mail. Workgroup members were asked to carefully scrutinize whether the determinants accurately and completely reflected their input as well as provide additional comments and suggestions. Their feedback was integrated into an adapted version of the framework by the first author, which was considered as the final framework version coming out of Phase 1, and which was used for the evaluation process in Phase 2.

### Results

#### Step 1.1

**Fig 2:**
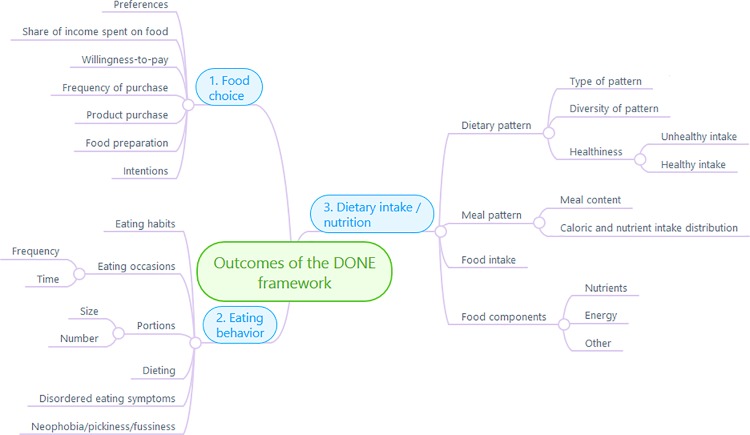
Taxonomy of outcomes of the DONE framework. Note: figure prepared using MindMeister.com.

The initial mind mapping procedure generated 145 distinct nutrition- and eating-related outcomes. The reduction and structuring of these outcomes by the workgroup leaders (MS and BR) resulted in a total of 37 outcomes, which was put forward to the workgroup. In the subsequent live discussion, this preliminary structure was further reduced to a final taxonomy. The final taxonomy consists of 34 outcomes grouped into three main sections (see Fig 2, reproduced from Stok et al., manuscript in preparation). These sections are: (1) food choice, encompassing outcomes preceding the actual consumption of food (e.g. preferences, intentions, purchase behaviors); (2) eating behavior, encompassing outcomes to do with the actual act of eating (e.g. frequency, amount, habits, dieting); and (3) dietary intake / nutrition, encompassing all outcomes related to *what* is consumed (e.g. healthy versus unhealthy intake, dietary patterns, food components). This taxonomy formed the basis for a common understanding within the workgroup and ensured shared representation of the outcomes that the determinants would be nominated for in the subsequent framework creation steps.

#### Step 1.2

**Fig 3:**
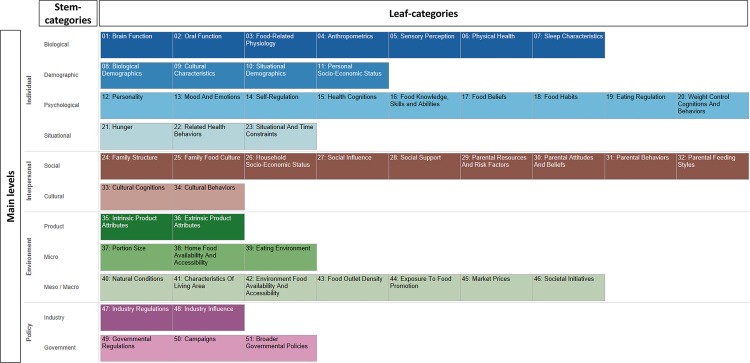
DONE framework categorization structure.

The generation and categorization of determinants resulted in a DONE framework comprising 441 determinants, of which 137 determinants (or 31%) occur in the framework twice (once for children and once for adults). Eighteen determinants uniquely influential in ethnic minority populations were discerned (three of which occur twice–once for children and once for adults). These determinants are structured into the framework like all other determinants, but are recognizable by the prefix EM. S1 Table lists all individual determinants currently included in the framework. Through the three Delphi consensus rounds, a structured categorization for the framework was agreed upon, which is depicted in Fig 3. Within the four main levels of socio-ecological influence, eleven stem-categories were discerned, and, within these stem-categories, a total of 51 leaf-categories were agreed upon. For example, two stem-categories (Social and Cultural) were discerned within the main level “Interpersonal”, and two leaf-categories (Cultural Cognitions and Cultural Behaviors) were distinguished within the “Cultural” stem-category. Table 2 provides a more detailed overview of all leaf-categories, including a brief explanation of each leaf-category and examples of determinants included in each leaf-category.

**Table 2:** Overview of leaf-categories with explanations and examples of determinants.

Level; stem-category	Leaf-category	Explanation	Examples of determinants
Individual; Biological	Brain Function	brain and brain functionality	dementia, orbito-frontal cortex volume
	Oral Function	oral system and oral functionality	chewing problems, wearing dentures
	Food-Related Physiology	physiological characteristics especially relevant for diet and nutrition that are not covered in the previous categories	food allergies, obesity-associated genes
	Anthropometrics	physical size and shape	BMI, birth weight
	Sensory Perception	sensory system and sensory perception	fat liking, taste preferences
	Physical Health	physical health status	medication use, chronic diseases
	Sleep Characteristics	sleep and sleeping patterns	chronotype, sleep duration
Individual; Demographic	Biological Demographics	(usually) innate demography	age, gender
	Cultural Characteristics	culturally-defined demography	nationality, ethnicity
	Situational Demographics	situationally defined demography	living arrangement, urban or rural dweller
	Personal Socio-Economic Status	socio-economic aspects of the individual	income, education
Individual; Psychological	Personality	personality traits and styles	self-esteem, personal values
	Mood And Emotions	affective states and stable moods	depressive symptomatology, positive emotions
	Self-Regulation	individual-difference traits concerned with controlling the self	impulsivity, self-control
	Health Cognitions	personal ideas and goals concerned with being healthy and eating healthily	health consciousness, healthy eating motivation
	Food Knowledge, Skills, and Abilities	personal resources relevant for diet and eating	nutrition knowledge, cooking skills
	Food Beliefs	personal thoughts and beliefs about food and eating	food ethics, trust in the food industry
	Food Habits	habits and routines around food consumption	habitual eating, willingness-to-pay
	Eating Regulation	psychological strategies for regulation of consumption	external eating, mindful eating
	Weight Control Cognitions And Behaviors	psychological aspects of weight control	body dissatisfaction, cognitive constraint
Individual; Situational	Hunger	situational occurrence of feeling hungry	hunger, food deprivation
	Related Health Behaviors	engagement in other health behaviors related to eating	alcohol consumption, television viewing
	Situational And Time Constraints	situational occurrences that impose constraints on consumption	access to a car, workload
Interpersonal; Social	Family Structure	composition and cohesion of the family / household	household size, family cohesion
	Family Food Culture	food culture existing in the family / household	household food processing, family food preferences
	Household Socio-Economic Status	socio-economic aspects of the family / household	household food security, household budget constraints
	Social Influence	diet- and eating-related influences from others in the environment	peer modeling, social norms
	Social Support	diet- and eating-related support from others in the environment	social ties, community recommendations
	Parental Resources And Risk Factors	parental resources and constraints relevant for diet and eating	parental time constraints, parental nutrition knowledge
	Parental Attitudes And Beliefs	parental thoughts and beliefs about food and eating	parental food risk aversion, parental trust in food distribution
	Parental Behaviors	parental food- and eating-related behaviors	parental food habits, parental frugality
	Parental Feeding Styles	how parents go about feeding their children	parental food restriction, parental pressure-to-eat
Interpersonal; Cultural	Cultural Cognitions	thoughts and beliefs related to one’s cultural background	cultural values, social role of food
	Cultural Behaviors	behaviors related to one’s cultural background	cultural food customs, religious rituals
Environmental; Product	Intrinsic Product Attributes	attributes intrinsic to the food product itself	product flavor, product texture
	Extrinsic Product Attributes	attributes extrinsic to the food product itself	product appearance, product price
Environmental; Micro	Portion Size	size of a food portion	portion size, visual cues to portion size
	Home Food Availability And Accessibility	availability and accessibility of food within the home	product visibility, food availability
	Eating Environment	the environment in which food is consumed	meal environment, enhanced eating environment
Environmental; Meso-Macro	Natural Conditions	natural conditions at the living location	weather, season
	Characteristics Of Living Area	the living environment	area deprivation, size of municipality
	Environment Food Availability And Accessibility	availability and accessibility in the environment	spatial distance food-consumer, neighborhood healthy food availability
	Food Outlet Density	density of food outlets in the environment	fast food outlet density, supermarket density
	Exposure To Food Promotion	presence of food promotion in the environment	exposure to food adverts, purchase prompts and food outlet
	Market Prices	cost of food	market prices, cost of a healthier basket
	Societal Initiatives	food- and eating-related social initiatives in the environment	community-supported agriculture programs, food-related NGO activity
Policy;Industry	Industry Regulations	guidelines and regulations for the food industry	nutritional composition regulations, portion-size regulations
	Industry Influence	exertion of influence by the food industry	Lobbying
Policy;Government	Governmental Regulations	food- and eating-related policies and regulations imposed by the government	food advertisement bans, subsidies for healthy food
	Campaigns	food- and eating-related governmental campaigns	educational campaigns for healthy foods, programs discouraging unhealthy eating
	Broader Governmental Policies	other relevant policies and regulations imposed by the government	immigrant-related policy, governmental health awareness

### Summary

A multiphase, multimethod approach was employed for the creation of the DONE framework. After mind mapping the outcomes for which determinants would be listed, five subgroups nominated determinants shaping these outcomes. Through multiple Delphi consensus rounds, the core workgroup integrated and structured the lists of determinants generated within each subgroup into an overarching framework. The framework was scrutinized by all members of the workgroup in a final Delphi consensus round, after which a version with 441 determinants, sorted into three layers of categorization and taking into account the role of different age groups, was agreed upon.

## Phase 2: Framework evaluation

The evaluation phase had two main aims. Firstly, we aimed to determine areas of priority for research by rating the framework’s determinants included on three different dimensions indicative of research priority [18,21]: modifiability, relationship strength, and population-level effect. Secondly, we aimed to assess the completeness of the framework (both in terms of categories and in terms of individual determinants) and to evaluate the usefulness of the framework for research and intervention. The framework was subsequently evaluated in two main steps; a graphical representation of the method is depicted in Fig 1.

The external experts who participated in the evaluation of the network were identified and recruited by the workgroup members involved in the framework creation. Experts could be from within DEDIPAC as long as they had not contributed to the creation of the DONE framework. More than 200 external and international experts were invited via e-mail to participate in both the rating and evaluation (which were separate parts of one large survey). The experts’ academic backgrounds and nationalities were diverse, as was the age group typically focused on in their research (see Table 1 for more details).

### Method

#### Step 2.1

In this step, determinants were rated on three dimensions to identify areas of priority for research. The first dimension that was rated was *modifiability*: the extent to which it “is possible to change the influence [of the determinant] in a healthful direction” [18]. Modifiability was rated on a three-point scale (1 = low; 2 = medium; 3 = high). The second dimension was *relationship strength*: the strength of the relation between determinant and outcome as judged by the rater. Relationship strength was rated on a two-point scale (1 = correlational; 2 = causal). The third dimension that was rated was *population-level effect*: the expected impact or reach of the determinant on eating behavior at the population-level, taking into account both association strength between determinant and individual behavior as well as prevalence of exposure to the determinant in the population. Population-level effect was rated on a three-point scale (1 = low; 2 = medium; 3 = high).

First, each member of the workgroup was asked to rate a subset of about 50 specific determinants taken from the framework on the three aforementioned dimensions, in order to identify priorities for research. They only received determinants related to the age group in which they had research expertise (i.e. workgroup members conducting research on nutrition in children received determinants from the children framework, et cetera). Ratings were provided by 59 members of the workgroup. Several workgroup members provided additional ratings (e.g. rating both a subset of adult determinants as well as a subset of child determinants). Each workgroup member received their subset of determinants via e-mail, completed their ratings, and sent the filled-out sheet back to a core workgroup member. Second, external experts were invited to rate the determinants on the same three dimensions via an online survey. The IRB of the University of Konstanz confirmed that the expert survey complies with the ethics guidelines of the university and with all national and international guidelines. By including ratings from external experts, we aimed to increase generalizability of the scoring system and thereby improve opportunities to draw conclusions regarding research priorities from the data. Furthermore, workgroup members’ ratings were found to vary substantially, and, by increasing the numbers of ratings, we aimed to improve validity of the average scores. Experts only received determinants related to the age group they indicated conducting research on (i.e. experts conducting research on nutrition in children received determinants from the children framework, et cetera). To minimalize burden on the external experts, each expert was asked to rate a subset of only ten determinants. A total of 123 external experts provided ratings. Both workgroup members and external experts could refrain from giving a rating if they felt they did not have sufficient knowledge to judge a specific determinant or dimension. For exact instructions given to the workgroup members and external experts regarding the ratings, please refer to the description in S1 Appendix.

Ratings from workgroup members and external experts were analyzed together. In addition to analyzing the three dimensions separately, we generated a ‘priority for research’ score including each of these three dimensions, allowing for an evaluation of overall priority. In order to equally weight each of the three dimensions, which were measured on different scales, the following formula was used: (modifiability rating / 3 + relationship strength rating / 2 + population-level effect rating / 3). Moreover, besides analyzing continuous rating results, we also categorized the average (i.e., across all raters) ratings per determinant and per dimension (see also Booth et al. [18]). Four categories were created for each of the three rating dimensions. For modifiability and population-level effect, measured on a three-point scale, the categories were low (1.00–1.49), moderate (1.50–1.99), substantial (2.00–2.49), and high (2.50–3.00). For relationship strength, measured on a two-point scale, the categories were low (1.00–1.24), moderate (1.25–1.49), substantial (1.50–1.74), and high (1.75–2.00).

#### Step 2.2

The same external experts who contributed to Step 2.1 were subsequently asked to take part in a second part of the online survey evaluating the DONE framework. A total of 113 experts completed the whole survey, and 3 additional experts completed part of the survey. The survey was conducted to receive input and feedback on the completeness and usefulness of the DONE framework from the larger scientific community and determine how people external to DEDIPAC evaluated the framework. The experts were asked to provide an assessment of the usefulness and comprehensiveness of the framework on five-point scales ranging from 1 = ‘not at all’ to 5 = ‘very much so’. Three open-ended questions invited additional feedback on the framework. Responses to the first open-ended question, “Can you evaluate the overall quality of the DONE framework?” were recoded into three categories (good–unable to judge–not good). Similarly, responses to the second open-ended question, “Would you consider using the DONE framework in your own research?” were recoded into three categories (yes–unsure–no). Responses to the third open-ended question, “Would you change the names of any of the categories in the framework?” were discussed extensively by the first two authors in order to determine their usefulness and validity.

### Results

#### Step 2.1

A total of 13,750 ratings were provided (by a total of 188 people) across all three rating dimensions, with a median of ten ratings available per determinant per dimension (*M* = 10.4, *SD* = 2.8, *range* = 3–28). Across all determinants, modifiability was rated at an average of 1.87 on a scale of 1 to 3. Relationship strength was rated, on average, at 1.43 on a scale of 1 to 2. Population-level effect was rated, on average, at 1.95 on a scale of 1 to 3. Finally, average overall priority (calculated by taking a weighted average of the three aforementioned ratings) was rated as 1.99 on a scale of 1 to 3. Average ratings on each dimension for each of the individual determinants, as well as the overall research priority rating, are provided in S1 Table; S1 Data provides all individual ratings. The number of ratings provided per determinant differed marginally significantly between the three dimensions, *F* (2,1320) = 2.59, *p* = .075. Post-hoc pairwise comparisons indicated that raters provided fewer relationship strength ratings (*M* = 10.1, *SD* = 3.1) than modifiability ratings (*M* = 10.6, *SD* = 2.7), *p* = .034 and also, marginally, than population-level effect ratings (*M* = 10.5, *SD* = 2.7), *p* = .077. The number of modifiability ratings and population-level effect ratings did not differ from each other, *p* = .772. Neither the number of ratings provided, nor the actual scores given, seemed to be moderated by type of rater (DEDIPAC workgroup member vs external expert) nor by rater background / expertise.

**Fig 4:**
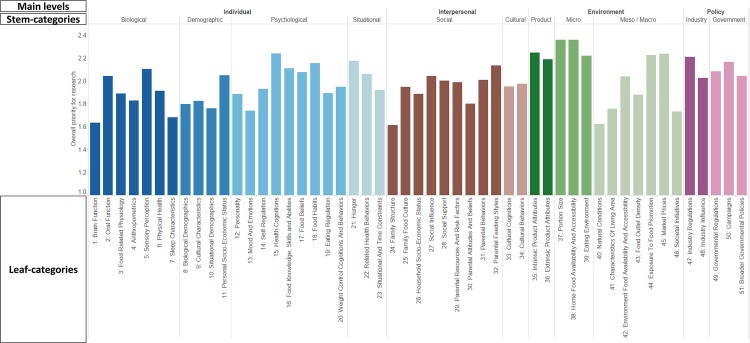
Average overall priority for research across determinants of the 51 leaf-categories in the DONE framework across age groups (children / adults) and across rater type (workgroup member / external expert).

**Fig 5:**
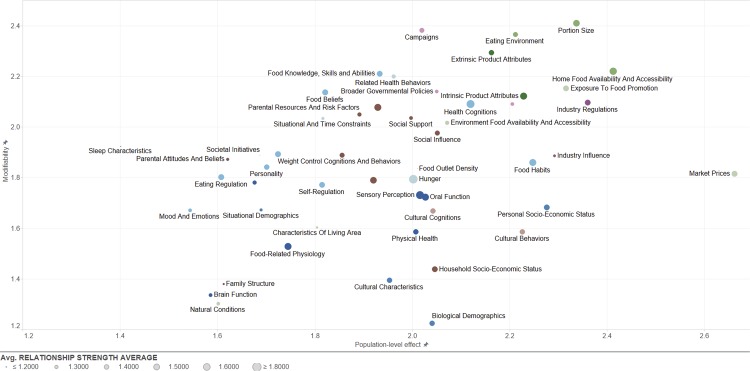
Average scores across determinants of the 51 leaf-categories in the DONE framework on modifiability, relationship strength, and population-level effect across age groups and across rater type. Sub-categories in the top-right corner that have larger circles can be considered as potentially important / influential leaf-categories as these sub-categories score highly on all three rating dimensions.

Results from the frequency analysis of categorized ratings, presented in Table 3, showed that there was a reasonable spread in all three ratings across the different categories. Most determinants were rated as having moderate (36.7%, 38.5%, and 41.7%) or substantial (43.1%, 32.7%, and 39.5%) modifiability, relationship strength, and population-level effect, respectively. Ratings were also aggregated per leaf-category. Fig 4 depicts the overall research priority for each of the 51 leaf-categories, while Fig 5 simultaneously depicts the scores for each leaf-category on the three separate rating dimensions. Figs 4 and 5 both depict the scores as provided across all age groups and including all determinants; in the online version of the framework, it is possible to individually view certain age groups or certain categories of determinants.

**Table 3:** Percentages of average (across workgroup members and external experts) determinant ratings falling into the categories low, moderate, substantial, and strong.

Category	Dimension		
	*Modifiability* (N = 441)	*Relationship strength* (N = 441)	*Population-level effect* (N = 441)
*Strong*	3.4%	8.8%	9.1%
*Substantial*	43.1%	32.7%	39.5%
*Moderate*	36.7%	38.5%	41.7%
*Low*	16.8%	20.0%	9.8%

Categorized scores of average (i.e., across all raters) ratings per determinant and per dimension (see also Booth et al. [18]). Four categories were created for each of the three rating dimensions. For modifiability and population-level effect, measured on a three-point scale, the categories were low (1.00–1.49), moderate (1.50–1.99), substantial (2.00–2.49), and high (2.50–3.00). For relationship strength, measured on a two-point scale, the categories were low (1.00–1.24), moderate (1.25–1.49), substantial (1.50–1.74), and high (1.75–2.00).

#### Step 2.2

The DONE framework was positively evaluated. The external experts rated both the usefulness and comprehensiveness of the DONE framework as high (*M* = 3.93, SD = 0.89 and *M* = 4.16, *SD* = 0.77, respectively on a scale of 1 to 5). The quality of the framework was judged to be “good” by 75% of the experts and as “not good” by 7%, while 18% indicated an inability to evaluate the quality. Moreover, 72% of the experts indicated that they would consider using the framework in their own line of research and/or teaching. A further 14% indicated not being sure at this point, while another 14% indicated not considering use of the framework. The evaluation data are available from S2 Data.

### Summary

The 441 determinants included in the DONE framework were rated by 188 different people on the dimensions of modifiability, relationship strength, and population-level effect. Results showed substantial to moderate average scores on each of these dimensions. Furthermore, quality, comprehensiveness, and usefulness of the framework were generally evaluated as satisfactory by a pool of interdisciplinary, international experts who had not been involved in the creation of the framework.

## Phase 3: Interactive framework updating

The DONE framework is conceptualized as a pioneering example of a “living”, evolving framework 2.0. As such, our intention is that determinants will continue to be added to the framework as more and more people contribute to the framework and as knowledge changes and increases. As the framework evolves, it is also possible to fine-tune the structure by adding new categories, merging old categories, or defining further levels of categorization. The third phase of the framework development process consisted of a first pilot round of such continued framework updating.

### Method

#### Step 3.1

The external experts (see Table 1 for more details) invited to rate the determinants and evaluate the framework were asked, in the same survey, to participate in the pilot round of updating the framework (for the exact survey items, please view S2 Appendix). Using a standardized, guided open-answer format, a total of 129 experts firstly provided the 5 factors they believed to be most influential for nutrition and eating. This question was designed to both assess whether or not our framework and conduct a first round of updating of the framework. After this, we provided the experts with a visualization of the DONE framework categories. Two additional open-answer format questions investigated whether experts believed the categorization structure of the DONE framework was complete and correct: “In your opinion, are there important categories missing in the framework?” and “Would you change the names of any of the categories in the framework?”. These two questions were answered by 119 and 117 experts, respectively. Finally, we asked the experts to fit each of their five most influential factors, which they had mentioned earlier, into the existing categories. We showed each of the five factors they had mentioned on the screen, along the visualization of the framework categories, and asked them to identify the most appropriate category in the framework for each factor. If the experts felt that a factor did not fit in any of the existing categories, they were asked to suggest an additional category to be added to the framework. This too was done using a standardized guided open-answer format and was meant to assess the completeness of the categories of our framework. A total of 112 experts provided responses on this item.

#### Step 3.2

In this final step, the DONE framework was visualized using Tableau Software version 9.1 (www.tableau.com). This software allows for an interactive depiction of the framework, including the priorities for research as identified in the ratings provided in Step 2.1. Within this visualization software, end users can filter the determinants to fit particular criteria, and thus specific research interests, and download the resulting data including rating information. Filters can be applied such that, for example, only determinants from certain levels, stem-categories or leaf-categories are depicted, or only determinants relevant for a certain age group or for ethnic minority populations. Various visualizations were created to depict different aspects of the framework. All interactive visualizations of the framework are freely available online on http://uni-konstanz.de/DONE.

### Results

#### Step 3.1

A total of 642 answers were provided in the first part of the survey, where experts were asked to name the five determinants of eating behavior that they considered most important. Two of the authors (MS and BR) systematically sorted through the answers to identify determinants not yet included in the DONE framework. Importantly, all but nine of the 642 determinants mentioned were already included in the framework, either verbatim or under another name (e.g. ‘having help from others’ was already included as ‘social support’), or a closely related concept was already included in the framework (e.g. for the determinant ‘previous experiences’ that was mentioned by an expert, the framework already includes the closely related determinants ‘food memories’ and ‘food familiarity’). In total, thus, the first framework updating round generated nine new determinants. All nine determinants could be placed into the already existing categorization structure of the framework, thus precluding the necessity to adapt the structure of the framework. These determinants are: genetic nutrient intolerances, hormones, mental health status, wellbeing, previous experience with disease, body weight perception, satiation, early exposure (children only), and sustainability awareness (adults only). These newly identified determinants are included in red font in S1 Table; they can also be recognized by the fact that ratings are not yet available for these determinants.

Similarly, two of the authors (MS and BR) systematically sorted through the answers provided to the questions regarding missing or misnamed categories. Forty-five percent of the experts provided a comment on the question about missing categories. Upon close inspection, however, most of these suggestions (42 responses, 78%) were found to suggest potential individual determinants that could be added to the framework rather than actual (stem- or leaf-) categories of determinants (please note that the experts were not shown the individual determinants; they only saw the categorization structure of the framework). These suggested determinants were carefully scrutinized, but all were found to already be included in the framework. Some suggestions also partially overlapped the new determinants described in the previous paragraphs. Twelve actual suggestions for new categories were provided. Upon careful consideration, it was decided that including any of the suggested categories would decrease the parsimony of the framework as each of the potentially new categories were already represented in the framework. Twenty-eight of the experts provided comments for the question about misnamed categories. All categories concerned were carefully scrutinized and, where deemed necessary, adjusted (following experts’ provided suggestions when these were available). For clarity’s sake, these changes have already been incorporated into the description of the framework throughout the entire article.

#### Step 3.2

**Fig 6:**
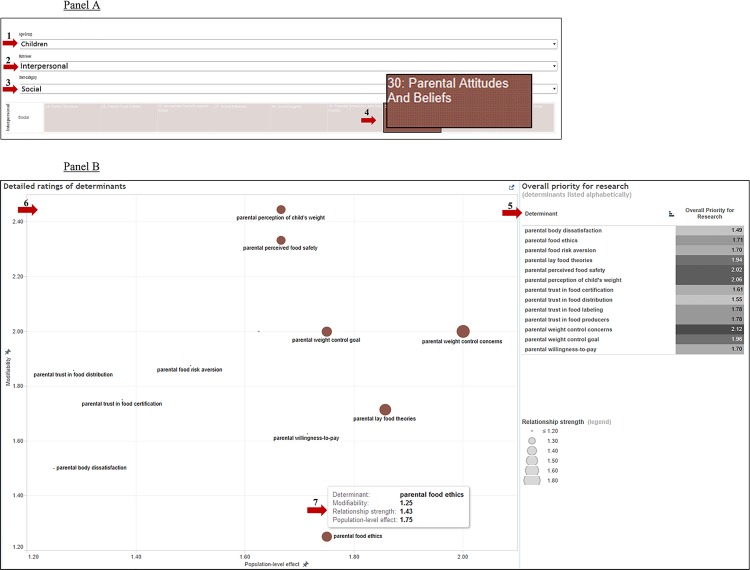
Storyboard detailing the interactive possibilities of the DONE framework using a concrete example of a potential research question.

Visualization of the framework resulted in a number of highly flexible, interactive representations of the determinants and ratings. To demonstrate the possibilities of this interactive visualization, we have created a sequence of visualizations based on an example of a potential concrete case: a researcher interested in designing an intervention aimed to promote healthier eating in children by intervening in parental attitudes and beliefs, who wants to know which specific determinants to target with his or her intervention. Starting from the basic DONE framework structure as depicted in Fig 3, Fig 6 shows the subsequent steps this policy maker could take. In Panel A, we show how the researcher would first pinpoint the specific age group (arrow 1), main level (arrow 2), stem-category (arrow 3), and leaf-category (arrow 4) in the DONE framework relevant to this aim. In Panel B, we show how the software then provides a list of the determinants included in this leaf-category, including overall priority for research scores (arrow 5) as well as a scatterplot showing the detailed ratings on all three dimensions for each determinant (arrow 6). By hovering over each determinant in the scatterplot (arrow 7), the researcher can see the exact rating scores on each dimension. Using this information, the researcher would then be able to decide which determinants might be most relevant to target with his or her intervention.

### Summary

A pilot updating round was conducted and resulted in the addition of nine new determinants to the DONE framework. Furthermore, external experts provided several suggestions for adding and renaming categories, several of which were incorporated after their validity was examined by two of the authors. Ultimately, our intention for the DONE framework is to use an online interactive platform to continue updating and evolving the framework beyond its initial publication. This is further elucidated in the Outlook and Conclusions section below.

## Outlook and conclusions

In this article, we have described the creation and evaluation of the interdisciplinary DONE framework, a dynamic, interactive framework 2.0 of the determinants of nutrition and eating. We have also described a pilot for continued updating of the framework, which is meant to continue beyond the publication of this article. In the creation phase, mind mapping, knowledge mapping, and several discussion and consensus rounds were employed to generate a comprehensive, systematically structured set of determinants of nutrition and eating across the lifespan. In the evaluation phase, priorities for research were determined by rating the determinants on the dimensions of modifiability, relationship strength, and population-level effect. Furthermore, the framework’s quality, usefulness, and comprehensiveness were empirically evaluated by external experts from different disciplines and countries. In the updating phase, a pilot confirmed the feasibility of the continued evolution of the framework by requesting additional input from external experts. Moreover, the framework was dynamically visualized and made freely available on the Internet.

### Application of the DONE framework: A perspective

With the publication of this article, the DONE framework is made available to the scientific and practical communities at large. Researchers, policy makers, and other end users can utilize the DONE framework 2.0 in a highly interactive and flexible way, as is demonstrated in the storyboard in Fig 6. Sub-models tailored to specific research questions can be created within the overarching framework and the relevant data (i.e. ratings of the relevant determinants) can be downloaded. Moreover, as the updating pilot showed, the framework is not static and allows for the continued evolution of the determinants and structure. This interactive approach allows extensions of the framework’s breadth and depth according to ongoing developments in research.

A core group of people involved in the creation of the framework will maintain and update the website on which the framework is available. Using a standard form, visitors can suggest new determinants and also specify in which category the new determinant should be placed (by either using an existing category or suggesting a new category). These suggestions will be appraised for validity and plausibility, and the framework will be updated in an event-based manner (when a certain number of new determinants have been suggested). Ratings for the new determinants will be obtained by asking every visitor to the website to provide such ratings. The rating form will also keep track of raters’ professions and areas of expertise. Visitors can also provide general feedback on the framework, for example by suggesting that certain ratings may be outdated or that important categories are still missing from the framework.

### Limitations

The DONE framework was created within a European research project. As such, the contribution from scientists outside Europe is extremely limited (several external experts came from Northern America, but this was only a minor proportion of the people involved in the framework). Involving additional people with different geographical backgrounds, but also from further varying scientific backgrounds, is thus an important future step for the further development of the framework. This is relevant both for the nomination of additional determinants (for example with regard to ethnic minorities, for whom eating context and influencing factors may be very different in other geographical areas), as well as for procuring additional ratings of the determinants. Our analyses showed large variability in ratings of modifiability, relationship strength and population-level effect. The more expert ratings we receive, the more reliable and valid the mean scores can be considered to be. Moreover, a major challenge for the future will be to introduce into the framework structural information regarding interrelations between the determinants. This is necessary in order to identify the moderators and mediators that modulate the direct impact of determinants. Finally, the DONE framework was evaluated as useful and, from a pilot updating round, seems to be quite complete. The true proof of the pudding, however, is of course in the eating: it is only through application and further refinement of the framework by researchers and public health professionals alike that the true value of the framework can be judged.

### Conclusion

Similar to the WEB 2.0, the web-based DONE framework 2.0 will enable users to create new content, comment on existing content, and share content with other users. A further perspective is that the web-based framework will facilitate interaction between researchers by reducing major technical barriers and enabling a more real-time development of the framework. Existing information can be re-used, modified, and added to growing databases of crowd-sourced knowledge open to a wider audience. This technological progress blurs the lines between the reception and production of new content. Ideally, the further development of the DONE framework would comprise continued evolution in the breadth and depth of the framework according to ongoing developments in research and inform novel developments in research and practice.

## Supporting information

S1 Appendix Instructions for supplying ratings of the determinants on modifiability, relationship strength, and population-level effect. (PDF)

S2 Appendix Questions to external experts for pilot updating round. (PDF)

S1 Table All determinants in the DONE framework, separated into the two main age groups (adults vs. children) and ordered by level, stem-category and leaf-category. Within each leaf-category, determinants are ordered by overall priority for research, from highest to lowest priority. Newly identified determinants (see Phase 3, Step 3.1) are identified in red font and always included first in a leaf-category. *Abbreviations*: OPR = Overall Priority for Research, Mod = Modifiability, RS = Relationship Strength, PLE = Population-Level Effect, SA = School-Aged.
(PDF)

S1 Data Individual ratings of the determinants in the DONE framework, including age group, level, stem-category and leaf-category. (XLSX)

S2 Data Data of the evaluation of the framework by the external experts. (XLSX)
