# Supplementary material for: The DONE framework: Creation, evaluation, and updating of an interdisciplinary, dynamic framework 2.0 of determinants of nutrition and eating
Source: PLoS One. 2017 Feb 2;12(2):e0171077. doi: 10.1371/journal.pone.0171077 (PMC5289713; doi:10.1371/journal.pone.0171077)
Supplement: S1 Appendix — (PDF) [file pone.0171077.s001.pdf]

The DONE framework: Creation, evaluation, and updating of an interdisciplinary, dynamic framework 2.0 of determinants of nutrition and eating

S1 Appendix

Appendix 1

Instructions for supplying ratings of the determinants on modifiability, relationship strength, and population-level effect

Note: This supporting file provides the exact instructions given to both workgroup members and external experts regarding the rating of the determinants on modifiability, relationship strength, and population-level effect. Workgroup members were invited to provide ratings via e-mail, allowing for slightly more extensive instructions than was the case with the external experts (who supplied ratings via an online survey). Importantly, the core content of the instructions is similar for both types of raters.

Instructions for partners

We would now like to proceed to the next stage of the framework, which consists of rating all the determinants on three dimensions:

1. Modifiability (ratings: 1 = difficult to modify, 2 = medium, 3 = easy to modify)
2. Relationship strength (ratings: 1 = correlational, 2 = causal)
3. Population-level effect (ratings: 1 = low effect, 2 = medium effect, 3 = high effect)

Definitions and instructions for rating each of these three dimensions are included below. In order to divide the work and keep the workload manageable for everybody, we propose that each partner rates a subset of the full set of determinants. Also attached to the email is an excel file with a subset of ±50 randomly selected determinants (file “subset […]”); these are the determinants we ask you to rate. Each partner receives a different subset, so as to receive as many diverse responses as possible.

Please note that we do not ask that you go into the literature to provide truly evidence-based ratings. We are interested on your intuitive and subjective thoughts, based on your expertise and experience. We know that you are not necessarily experts on all the determinants we ask you to rate, but that is by design: we are aiming to accumulate a cross-disciplinary perspective on all the determinants. If there is a determinant you really do not know, you can leave this one empty and indicate this in the comments column.

Assigning level of modifiability in the DONE framework
A determinant can be considered modifiable if “it is possible to change the influence [of the determinant] in a healthful direction” (Booth et al., 2001). The determinant should thus be “susceptible to change through either broad-based individual choices or public policy choices” (Johnson-Taylor & Everhart, 2006). We want to add to this that the change should be able to take place within a reasonable time frame (i.e. it should not be observable only after many years).

Please rate modifiability of each determinant on a 3-level scale:

1 = difficult to modify
2 = medium
3 = easy to modify

Assigning relationship strength in the DONE framework
For assessing the type of relationship between determinant and outcome, we started off with a rather complex coding scheme (see figures below). In practice, we have experienced that this scheme is too complicated. Therefore, in our final framework, we will first of all include only direct relations (so we will not include mediators, moderators, and confounders). We ask you to choose for each variable the highest level for which there is evidence (which is somewhat conclusive, i.e. identifying a variable as a determinant should not be based on just one study showing a causal relation whereas all other available studies only show an association).

Please rate relationship strength of each determinant on a 2-level scale:
1 = correlational: there is an association, but no inference of causality
2 = causal: there is a causal relation

Assigning population-level effect in the DONE model
Population-level effect is the expected impact of the determinant on eating behavior at the population-level. Your assessment of this should take into account both the strength of association between the determinant and individual behavior as well as the prevalence of exposure to the determinant in the population. The idea is to reach an evaluation of the potential reach or impact of the determinant on a large (population-level) scale.

Please rate population-level effect of each determinant on a 3-level scale:
1 = low population-level effect
2 = medium population-level effect
3 = high population-level effect

<u>Instructions for external experts</u>
We are interested in your expert opinion, based on your personal expertise and experience. We know that you are not necessarily experts on all the factors we ask you to rate, but that is by design: we are aiming to accumulate a cross-disciplinary perspective on all the factors.
The dimensions on which you will rate the factors are:

1: modifiability. Is the factor susceptible to change (through either individual choices, communal choices or public policy choices)? The answering options here are easy to modify – medium – difficult to modify.

2: relationship strength. Based on your knowledge of the available evidence, is the factor only associated with dietary behavior or is there a causal relationship? The answering options here are correlational – causal.

3: population-level effect. This refers to the expected impact of the factor on dietary behavior at the population level. Your assessment should take into account both the strength of association between the factor and dietary behavior as well as the prevalence of exposure to the factor in the population. The answering options here are low effect – medium effect – high effect.
