# Supplementary material for: The DONE framework: Creation, evaluation, and updating of an interdisciplinary, dynamic framework 2.0 of determinants of nutrition and eating
Source: PLoS One. 2017 Feb 2;12(2):e0171077. doi: 10.1371/journal.pone.0171077 (PMC5289713; doi:10.1371/journal.pone.0171077)
Supplement: S2 Appendix — (PDF) [file pone.0171077.s002.pdf]

The DONE framework: Creation, evaluation, and updating of an interdisciplinary, dynamic framework 2.0 of determinants of nutrition and eating

S2 Appendix

Appendix 2

Questions to external experts for pilot updating round

The DONE framework has been developed within the DEDIPAC project. Our aim is to include all factors associated with dietary behavior, nutrition and food choice. In order to judge whether we have been successful in including the most important factors, we would like to ask you to list below the five factors that you believe are the most important factors of dietary behavior, nutrition and food choice.

1: ____

2: ____

3: ____

4: ____

5: ____

Finally, we would like to show you the current structure of the DONE framework. The factors in the DONE framework are organized on three levels of increasing specificity. The figure below shows you these levels. There are thus no individual factors in this figure, only the overall structure of the categories to which all the factors belong.

We would like to ask you to browse through the categories and respond to some questions about the structure of the framework. The categories are presented here and will also be shown on each of the next pages accompanying the questions.

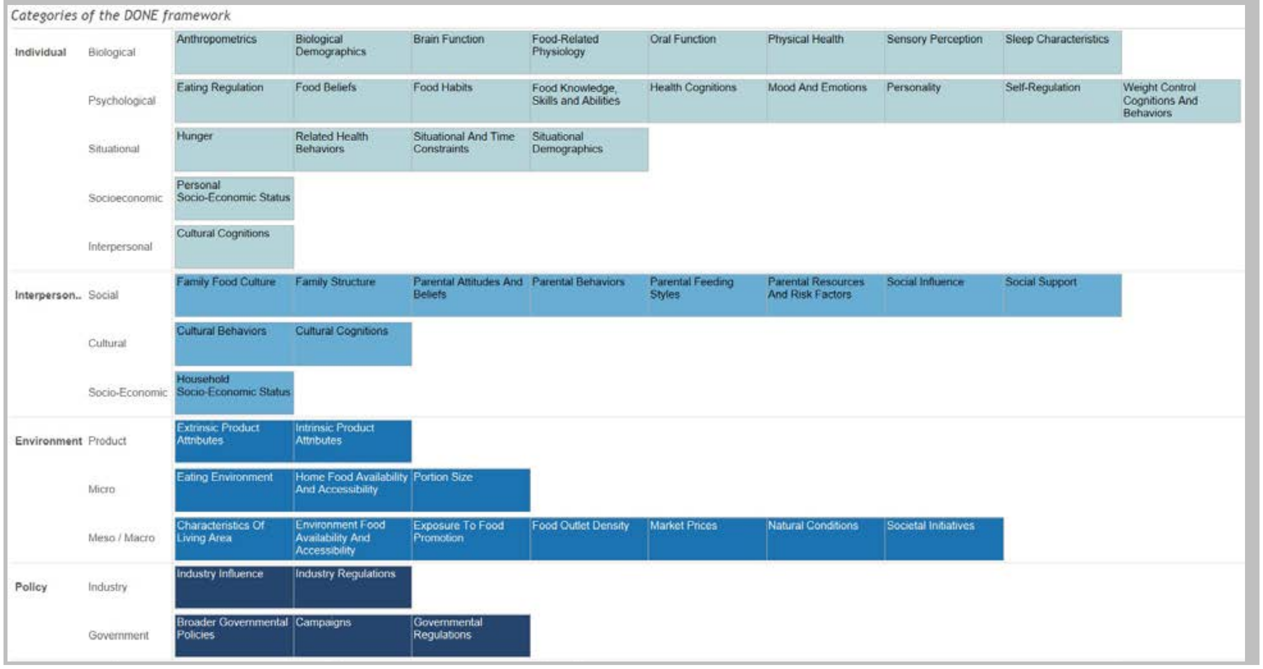

In your opinion, are there important categories missing in the framework?
[Open answer format]

Would you change the names of any of the categories in the framework?
[Open answer format]

We would also like to ask you to categorize the five factors that you indicated as important earlier in the survey. In which category would you place each of these five factors? If there is no fitting category for your factor in the framework yet, please write the name of a category you think should be included in the DONE framework to which your factor would fit.

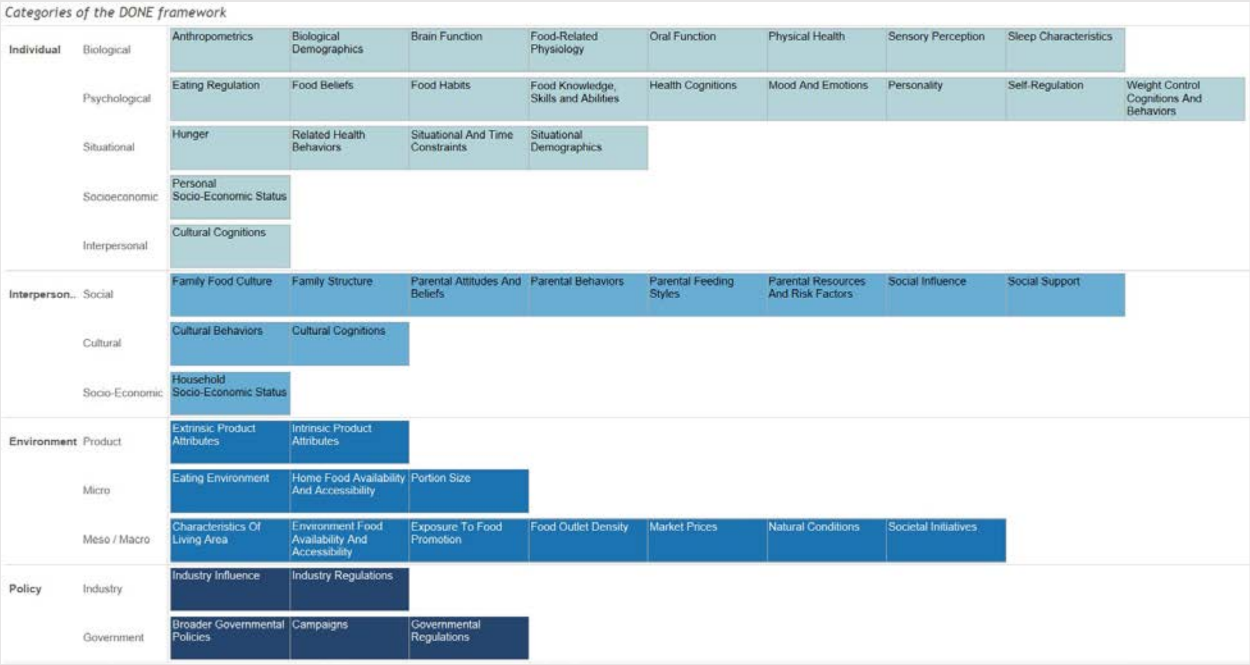

[1st factor mentioned]: ____
[2nd factor mentioned]: ____
[3rd factor mentioned]: ____
[4th factor mentioned]: ____
[5th factor mentioned]: ____
