# Supplementary material for: The DONE framework: Creation, evaluation, and updating of an interdisciplinary, dynamic framework 2.0 of determinants of nutrition and eating
Source: PLoS One. 2017 Feb 2;12(2):e0171077. doi: 10.1371/journal.pone.0171077 (PMC5289713; doi:10.1371/journal.pone.0171077)
Supplement: S1 Table — Within each leaf-category, determinants are ordered by overall priority for research, from highest to lowest priority. Newly identified determinants (see Phase 3, Step 3.1) are identified in red font and always included first in a leaf-category. Abbreviations: OPR = Overall Priority for Research, Mod = Modifiability, RS = Relationship Strength, PLE = Population-Level Effect, SA = School-Aged. (PDF) [file pone.0171077.s003.pdf]

The DONE framework: Creation, evaluation, and updating of an interdisciplinary, dynamic framework 2.0 of determinants of nutrition and eating

S1 Table

Determinant	Age Group	Main level	Stem-category	Leaf-category	OPR	Mod	RS	PLE
mental abilities	Adults	Individual	Biological	Brain Function	1.80	1.55	1.36	1.82
dementia	Elderly	Individual	Biological	Brain Function	1.66	1.00	1.40	1.87
orbitofrontal cortex volume	Adults	Individual	Biological	Brain Function	1.54	1.40	1.11	1.56
mental retardation	Adults	Individual	Biological	Brain Function	1.53	1.40	1.40	1.10
dental deficiency	Elderly	Individual	Biological	Oral Function	2.21	2.13	1.63	2.06
chewing problems	Elderly	Individual	Biological	Oral Function	2.20	1.80	1.73	2.20
wearing dentures	Elderly	Individual	Biological	Oral Function	2.07	1.69	1.56	2.19
pain when eating	Elderly	Individual	Biological	Oral Function	2.00	1.81	1.63	1.75
number of natural teeth	Elderly	Individual	Biological	Oral Function	1.74	1.19	1.40	1.93
genetic nutrient intolerances	Adults	Individual	Biological	Food-Related Physiology	N.A.	N.A.	N.A.	N.A.
hormones	Adults	Individual	Biological	Food-Related Physiology	N.A.	N.A.	N.A.	N.A.
food allergies	Adults	Individual	Biological	Food-Related Physiology	2.19	1.92	1.92	1.77
physical limitations in food preparation	Elderly	Individual	Biological	Food-Related Physiology	2.09	1.63	1.69	2.13
physical limitations in consumption	Elderly	Individual	Biological	Food-Related Physiology	2.09	1.53	1.73	2.13
taste sensitivity	Adults	Individual	Biological	Food-Related Physiology	2.07	2.00	1.58	1.83
anorexia of aging	Elderly	Individual	Biological	Food-Related Physiology	2.06	1.44	1.75	2.13
appetite	Adults	Individual	Biological	Food-Related Physiology	1.92	1.92	1.50	1.58
gut microbiota	Adults	Individual	Biological	Food-Related Physiology	1.87	2.15	1.23	1.62
diminished sense of taste	Elderly	Individual	Biological	Food-Related Physiology	1.80	1.20	1.60	1.80
odor threshold	Elderly	Individual	Biological	Food-Related Physiology	1.73	1.31	1.38	1.81
obesity-associated genes	Adults	Individual	Biological	Food-Related Physiology	1.59	1.36	1.40	1.30
weight	Adults	Individual	Biological	Anthropometrics	2.06	2.18	1.45	1.82
BMI	Adults	Individual	Biological	Anthropometrics	2.00	2.00	1.29	2.07
body composition	Adults	Individual	Biological	Anthropometrics	1.95	2.14	1.29	1.79
central obesity	Adults	Individual	Biological	Anthropometrics	1.90	1.75	1.42	1.83
muscle mass	Adults	Individual	Biological	Anthropometrics	1.88	2.00	1.45	1.45
food liking	Adults	Individual	Biological	Sensory Perception	2.29	1.92	1.92	2.08
food preferences	Adults	Individual	Biological	Sensory Perception	2.29	1.91	1.73	2.36
fat liking	Adults	Individual	Biological	Sensory Perception	2.26	1.92	1.69	2.31
learned taste preferences	Adults	Individual	Biological	Sensory Perception	2.22	1.85	1.77	2.15
taste aversions	Adults	Individual	Biological	Sensory Perception	2.04	1.83	1.75	1.67
food wanting	Adults	Individual	Biological	Sensory Perception	1.95	1.70	1.50	1.90
preference for bitter taste	Adults	Individual	Biological	Sensory Perception	1.92	1.58	1.83	1.42
biological taste preferences	Adults	Individual	Biological	Sensory Perception	1.83	1.69	1.46	1.62
mental health status	Adults	Individual	Biological	Physical Health	N.A.	N.A.	N.A.	N.A.
health status	Adults	Individual	Biological	Physical Health	2.18	1.92	1.58	2.25
physical health status	Elderly	Individual	Biological	Physical Health	2.11	1.88	1.44	2.31
medication use	Adults	Individual	Biological	Physical Health	2.08	1.96	1.44	2.11
chronic diseases	Adults	Individual	Biological	Physical Health	1.98	1.86	1.29	2.14
intensity level of required care	Elderly	Individual	Biological	Physical Health	1.96	1.53	1.33	2.33
frailty	Elderly	Individual	Biological	Physical Health	1.80	1.47	1.20	2.13
sleep duration	Adults	Individual	Biological	Sleep Characteristics	1.94	2.36	1.27	1.55
sleep difficulties	Adults	Individual	Biological	Sleep Characteristics	1.79	1.93	1.14	1.71
chronotype	Adults	Individual	Biological	Sleep Characteristics	1.65	1.58	1.25	1.50
midpoint of sleep	Adults	Individual	Biological	Sleep Characteristics	1.62	2.00	1.10	1.20
age	Adults	Individual	Demographic	Biological Demographics	2.04	1.57	1.50	2.29
sex	Adults	Individual	Demographic	Biological Demographics	1.87	1.30	1.55	2.00
religion	Adults	Individual	Demographic	Cultural Characteristics	2.08	1.90	1.50	2.10
cultural identity	Adults	Individual	Demographic	Cultural Characteristics	1.95	1.70	1.50	1.90
ethnicity	Adults	Individual	Demographic	Cultural Characteristics	1.91	1.67	1.55	1.75
nationality	Adults	Individual	Demographic	Cultural Characteristics	1.73	1.75	1.08	1.82
living alone	Elderly	Individual	Demographic	Situational Demographics	2.15	1.81	1.50	2.38
place of residence	Adults	Individual	Demographic	Situational Demographics	1.95	1.85	1.38	1.92
being widowed	Elderly	Individual	Demographic	Situational Demographics	1.82	1.25	1.31	2.25
living arrangement	Adults	Individual	Demographic	Situational Demographics	1.74	1.91	1.18	1.55
urban or rural dweller	Adults	Individual	Demographic	Situational Demographics	1.74	1.56	1.33	1.67
EM competency in host language	Adults	Individual	Demographic	Situational Demographics	1.67	2.00	1.17	1.25
EM length of stay in host country	Adults	Individual	Demographic	Situational Demographics	1.67	1.67	1.11	1.67
EM age at migration	Adults	Individual	Demographic	Situational Demographics	1.60	1.58	1.25	1.33
level of education	Adults	Individual	Demographic	Personal Socio-Economic Status	2.31	1.92	1.54	2.69
low income	Adults	Individual	Demographic	Personal Socio-Economic Status	2.19	1.55	1.55	2.70
income	Adults	Individual	Demographic	Personal Socio-Economic Status	2.15	2.08	1.38	2.31

Determinant	Age Group	Main level	Stem-category	Leaf-category	OPR	Mod	RS	PLE
socio-economic status	Adults	Individual	Demographic	Personal Socio-Economic Status	2.06	1.92	1.33	2.25
food insecurity	Adults	Individual	Demographic	Personal Socio-Economic Status	2.03	1.55	1.64	2.09
occupation	Adults	Individual	Demographic	Personal Socio-Economic Status	1.80	1.77	1.31	1.67
personal values	Adults	Individual	Psychological	Personality	2.06	2.08	1.50	1.83
self-efficacy	Adults	Individual	Psychological	Personality	2.05	1.93	1.57	1.86
personality traits	Adults	Individual	Psychological	Personality	1.97	1.83	1.64	1.64
self-esteem	Adults	Individual	Psychological	Personality	1.83	2.08	1.15	1.69
distractibility when eating	Adults	Individual	Psychological	Personality	1.79	2.07	1.27	1.40
wellbeing	Adults	Individual	Psychological	Mood And Emotions	N.A.	N.A.	N.A.	N.A.
positive emotions	Adults	Individual	Psychological	Mood And Emotions	1.95	1.67	1.58	1.82
mood	Adults	Individual	Psychological	Mood And Emotions	1.78	1.90	1.27	1.55
negative emotions	Adults	Individual	Psychological	Mood And Emotions	1.69	1.31	1.46	1.58
EM stress about migration	Adults	Individual	Psychological	Mood And Emotions	1.63	2.00	1.11	1.22
depressive symptomatology	Adults	Individual	Psychological	Mood And Emotions	1.63	1.62	1.31	1.31
self-regulation skills	Adults	Individual	Psychological	Self-Regulation	2.28	1.92	1.83	2.17
self-control	Adults	Individual	Psychological	Self-Regulation	2.18	2.00	1.69	2.00
executive functioning	Adults	Individual	Psychological	Self-Regulation	2.02	2.00	1.57	1.71
future time orientation	Adults	Individual	Psychological	Self-Regulation	1.98	2.00	1.38	1.88
emotion suppression	Adults	Individual	Psychological	Self-Regulation	1.96	1.92	1.62	1.54
impulsivity	Adults	Individual	Psychological	Self-Regulation	1.74	1.64	1.36	1.55
previous experience with disease	Adults	Individual	Psychological	Health Cognitions	N.A.	N.A.	N.A.	N.A.
health consciousness	Adults	Individual	Psychological	Health Cognitions	2.48	2.27	1.82	2.45
health concerns	Adults	Individual	Psychological	Health Cognitions	2.32	2.00	1.75	2.33
healthy eating motivation	Adults	Individual	Psychological	Health Cognitions	2.28	2.00	1.85	2.08
healthy eating intention	Adults	Individual	Psychological	Health Cognitions	2.18	2.18	1.70	1.80
perceived physical health	Elderly	Individual	Psychological	Health Cognitions	1.94	2.00	1.27	1.93
nutrition self-efficacy	Adults	Individual	Psychological	Food Knowledge, Skills and Abilities	2.38	2.40	1.70	2.20
dietary knowledge	Adults	Individual	Psychological	Food Knowledge, Skills and Abilities	2.38	2.54	1.57	2.25
food knowledge	Adults	Individual	Psychological	Food Knowledge, Skills and Abilities	2.35	2.33	1.75	2.08
food familiarity	Adults	Individual	Psychological	Food Knowledge, Skills and Abilities	2.28	2.17	1.64	2.22
nutrition knowledge	Adults	Individual	Psychological	Food Knowledge, Skills and Abilities	2.15	2.42	1.58	1.67
cooking skills	Adults	Individual	Psychological	Food Knowledge, Skills and Abilities	2.14	2.54	1.31	1.92
awareness of food consumed	Adults	Individual	Psychological	Food Knowledge, Skills and Abilities	2.00	2.38	1.31	1.67
food memories	Adults	Individual	Psychological	Food Knowledge, Skills and Abilities	1.96	1.83	1.58	1.67
memory for food adverts	Adults	Individual	Psychological	Food Knowledge, Skills and Abilities	1.91	2.00	1.15	2.00
perceived barrier of food price	Adults	Individual	Psychological	Food Beliefs	2.29	2.31	1.67	2.08
lay food beliefs	Adults	Individual	Psychological	Food Beliefs	2.26	2.09	1.73	2.09
perceived food safety	Adults	Individual	Psychological	Food Beliefs	2.22	2.33	1.67	1.83
perceived benefit of diet quality	Adults	Individual	Psychological	Food Beliefs	2.18	2.38	1.54	1.85
food ethics	Adults	Individual	Psychological	Food Beliefs	2.12	2.00	1.70	1.80
trust in the food industry	Adults	Individual	Psychological	Food Beliefs	2.05	2.45	1.36	1.64
food concerns	Adults	Individual	Psychological	Food Beliefs	2.04	2.08	1.58	1.67
food involvement	Adults	Individual	Psychological	Food Beliefs	2.03	2.10	1.40	1.90
food enjoyment	Adults	Individual	Psychological	Food Beliefs	2.01	1.83	1.58	1.83
attitudes toward sustainable foods	Adults	Individual	Psychological	Food Beliefs	1.99	2.17	1.42	1.67
food risk aversion	Adults	Individual	Psychological	Food Beliefs	1.90	2.20	1.40	1.40
habitual eating	Adults	Individual	Psychological	Food Habits	2.45	2.00	1.70	2.80
past eating behavior	Adults	Individual	Psychological	Food Habits	2.27	1.92	1.62	2.46
eating routines	Adults	Individual	Psychological	Food Habits	2.25	2.07	1.64	2.21
willingness-to-pay	Adults	Individual	Psychological	Food Habits	2.13	2.00	1.25	2.50
mindfulness	Adults	Individual	Psychological	Eating Regulation	2.15	2.08	1.75	1.75
mindful eating	Adults	Individual	Psychological	Eating Regulation	2.06	2.25	1.67	1.42
intuitive eating	Adults	Individual	Psychological	Eating Regulation	2.00	1.86	1.71	1.57
food selectivity	Adults	Individual	Psychological	Eating Regulation	1.97	1.92	1.50	1.75
neophobia	Adults	Individual	Psychological	Eating Regulation	1.94	1.82	1.64	1.55
external eating	Adults	Individual	Psychological	Eating Regulation	1.85	1.64	1.45	1.73
emotional eating	Adults	Individual	Psychological	Eating Regulation	1.85	1.58	1.58	1.58
body weight perception	Adults	Individual	Psychological	Weight Control Cognitions And Behaviors	N.A.	N.A.	N.A.	N.A.
weight loss intention	Adults	Individual	Psychological	Weight Control Cognitions And Behaviors	2.29	2.18	1.73	2.09
restrained eating	Adults	Individual	Psychological	Weight Control Cognitions And Behaviors	2.22	1.92	1.77	2.08
eating in the absence of hunger	Adults	Individual	Psychological	Weight Control Cognitions And Behaviors	2.15	2.31	1.54	1.85
weight control goal	Adults	Individual	Psychological	Weight Control Cognitions And Behaviors	2.10	2.08	1.58	1.83

Determinant	Age Group	Main level	Stem-category	Leaf-category	OPR	Mod	RS	PLE
weight control concerns	Adults	Individual	Psychological	Weight Control Cognitions And Behaviors	2.07	2.10	1.40	2.00
body dissatisfaction	Adults	Individual	Psychological	Weight Control Cognitions And Behaviors	1.85	2.00	1.36	1.50
cognitive restraint	Adults	Individual	Psychological	Weight Control Cognitions And Behaviors	1.72	1.77	1.21	1.57
disinhibition	Adults	Individual	Psychological	Weight Control Cognitions And Behaviors	1.65	1.82	1.18	1.36
satiation	Adults	Individual	Situational	Hunger	N.A.	N.A.	N.A.	N.A.
food deprivation	Adults	Individual	Situational	Hunger	2.17	1.92	1.77	1.92
hunger	Adults	Individual	Situational	Hunger	2.11	2.00	1.67	1.83
physical activity level	Adults	Individual	Situational	Related Health Behaviors	2.22	2.25	1.50	2.17
frequency of television viewing	Adults	Individual	Situational	Related Health Behaviors	2.07	2.17	1.25	2.17
alcohol consumption	Adults	Individual	Situational	Related Health Behaviors	2.01	2.36	1.21	1.85
smoking	Adults	Individual	Situational	Related Health Behaviors	2.00	2.10	1.40	1.80
individual food processing (cooking)	Adults	Individual	Situational	Related Health Behaviors	1.86	2.17	1.17	1.67
individual food production (growing)	Adults	Individual	Situational	Related Health Behaviors	1.75	2.00	1.10	1.60
infrequent care	Elderly	Individual	Situational	Situational And Time Constraints	2.22	2.42	1.33	2.25
kitchen facilities	Elderly	Individual	Situational	Situational And Time Constraints	2.06	2.40	1.27	1.87
perceived stress	Adults	Individual	Situational	Situational And Time Constraints	1.97	1.85	1.38	2.00
workload	Adults	Individual	Situational	Situational And Time Constraints	1.92	1.83	1.33	1.92
daily rhythm/structure	Adults	Individual	Situational	Situational And Time Constraints	1.90	2.15	1.23	1.69
work-life balance	Adults	Individual	Situational	Situational And Time Constraints	1.85	1.92	1.25	1.75
access to a car	Adults	Individual	Situational	Situational And Time Constraints	1.77	2.15	1.23	1.31
caregiving role	Adults	Interpersonal	Social	Family Structure	1.94	1.92	1.31	1.92
household size	Adults	Interpersonal	Social	Family Structure	1.86	1.75	1.33	1.83
household food processing (cooking)	Adults	Interpersonal	Social	Family Food Culture	2.33	2.36	1.45	2.45
household food production (growing)	Adults	Interpersonal	Social	Family Food Culture	1.81	1.92	1.31	1.54
household income	Adults	Interpersonal	Social	Household Socio-Economic Status	2.40	1.92	1.77	2.62
household socio-economic status	Adults	Interpersonal	Social	Household Socio-Economic Status	2.28	1.83	1.67	2.50
household budget constraints	Adults	Interpersonal	Social	Household Socio-Economic Status	2.21	1.91	1.64	2.27
household food security	Adults	Interpersonal	Social	Household Socio-Economic Status	2.08	1.92	1.50	2.08
social relationships	Adults	Interpersonal	Social	Social Influence	2.35	2.17	1.58	2.50
social norms	Adults	Interpersonal	Social	Social Influence	2.24	1.92	1.42	2.67
peer modeling	Adults	Interpersonal	Social	Social Influence	2.21	2.25	1.42	2.25
eating occasion	Adults	Interpersonal	Social	Social Influence	1.99	2.21	1.43	1.62
perceived important other's dieting	Adults	Interpersonal	Social	Social Influence	1.98	2.18	1.36	1.73
presence of others	Adults	Interpersonal	Social	Social Influence	1.97	1.92	1.38	1.92
social facilitation for dieting	Adults	Interpersonal	Social	Social Influence	1.96	1.92	1.42	1.83
decisional power in groups	Adults	Interpersonal	Social	Social Influence	1.81	2.00	1.17	1.67
EM inter-generational influences on diet	Adults	Interpersonal	Social	Social Influence	1.67	1.89	1.11	1.44
social support	Adults	Interpersonal	Social	Social Support	2.31	2.00	1.50	2.67
social ties	Adults	Interpersonal	Social	Social Support	2.13	2.17	1.42	2.08
frequency of social contacts	Adults	Interpersonal	Social	Social Support	2.06	2.38	1.17	2.04
community recommendations	Adults	Interpersonal	Social	Social Support	1.94	1.93	1.27	2.00
salesperson recommendations	Adults	Interpersonal	Social	Social Support	1.94	1.92	1.46	1.69
social role of food	Adults	Interpersonal	Cultural	Cultural Cognitions	2.15	2.00	1.50	2.20
cultural norms	Adults	Interpersonal	Cultural	Cultural Cognitions	2.08	1.71	1.50	2.29
cultural beliefs	Adults	Interpersonal	Cultural	Cultural Cognitions	2.04	1.73	1.47	2.20
cultural values	Adults	Interpersonal	Cultural	Cultural Cognitions	2.00	1.62	1.38	2.31
EM familiarization with host-country foods	Adults	Interpersonal	Cultural	Cultural Cognitions	1.88	2.09	1.27	1.64
EM status of traditional vs convience foods	Adults	Interpersonal	Cultural	Cultural Cognitions	1.83	2.13	1.25	1.50
EM perception of host culture	Adults	Interpersonal	Cultural	Cultural Cognitions	1.79	2.00	1.20	1.56
EM preferences for larger body size	Adults	Interpersonal	Cultural	Cultural Cognitions	1.56	1.89	1.11	1.11
cultural food habits	Adults	Interpersonal	Cultural	Cultural Behaviors	2.29	1.82	1.73	2.45
cultural traditions	Adults	Interpersonal	Cultural	Cultural Behaviors	2.14	1.64	1.57	2.43
religious rituals	Adults	Interpersonal	Cultural	Cultural Behaviors	2.14	1.73	1.55	2.36
cultural food customs	Adults	Interpersonal	Cultural	Cultural Behaviors	2.04	1.83	1.25	2.42
EM conformity to tradition	Adults	Interpersonal	Cultural	Cultural Behaviors	2.01	1.92	1.42	2.00
EM level of acculturation	Adults	Interpersonal	Cultural	Cultural Behaviors	1.81	2.11	1.11	1.67
sensory appeal	Adults	Environment	Product	Intrinsic Product Attributes	2.62	2.38	1.85	2.69
product taste	Adults	Environment	Product	Intrinsic Product Attributes	2.45	2.14	1.79	2.54
nutritional composition	Adults	Environment	Product	Intrinsic Product Attributes	2.44	2.33	1.75	2.36
product flavor	Adults	Environment	Product	Intrinsic Product Attributes	2.38	2.00	1.77	2.50
product texture	Adults	Environment	Product	Intrinsic Product Attributes	2.17	2.00	1.62	2.08
natural content of product	Adults	Environment	Product	Intrinsic Product Attributes	1.89	1.82	1.36	1.80

Determinant	Age Group	Main level	Stem-category	Leaf-category	OPR	Mod	RS	PLE
price	Adults	Environment	Product	Extrinsic Product Attributes	2.50	2.31	1.69	2.67
product convenience	Adults	Environment	Product	Extrinsic Product Attributes	2.32	2.15	1.54	2.50
product variety	Adults	Environment	Product	Extrinsic Product Attributes	2.30	2.25	1.58	2.27
package size	Adults	Environment	Product	Extrinsic Product Attributes	2.26	2.33	1.53	2.14
calorie labelling	Adults	Environment	Product	Extrinsic Product Attributes	2.20	2.71	1.21	2.07
nutritional information	Adults	Environment	Product	Extrinsic Product Attributes	2.16	2.33	1.50	1.91
product appearance	Adults	Environment	Product	Extrinsic Product Attributes	2.15	2.08	1.46	2.17
food labeling	Adults	Environment	Product	Extrinsic Product Attributes	2.13	2.23	1.38	2.08
product packaging	Adults	Environment	Product	Extrinsic Product Attributes	2.06	2.31	1.31	1.92
package color	Adults	Environment	Product	Extrinsic Product Attributes	2.01	2.25	1.25	1.91
product brand	Adults	Environment	Product	Extrinsic Product Attributes	1.93	1.50	1.57	1.93
portion size	Adults	Environment	Micro	Portion Size	2.55	2.45	1.82	2.45
visual cues to portion size	Adults	Environment	Micro	Portion Size	2.13	2.56	1.22	2.00
food accessibility	Adults	Environment	Micro	Home Food Availability And Accessibility	2.40	2.08	1.75	2.50
food availability	Adults	Environment	Micro	Home Food Availability And Accessibility	2.38	2.08	1.75	2.42
product visibility	Adults	Environment	Micro	Home Food Availability And Accessibility	2.04	2.15	1.31	2.00
enhanced eating environment	Elderly	Environment	Micro	Eating Environment	2.32	2.56	1.44	2.25
meal environment	Adults	Environment	Micro	Eating Environment	2.14	2.09	1.36	2.27
season	Adults	Environment	Meso / Macro	Natural Conditions	1.96	1.42	1.54	2.15
time of day	Adults	Environment	Meso / Macro	Natural Conditions	1.74	1.55	1.33	1.67
weather	Adults	Environment	Meso / Macro	Natural Conditions	1.55	1.60	1.18	1.27
globalization	Adults	Environment	Meso / Macro	Characteristics Of Living Area	2.05	2.09	1.18	2.27
geographical variation	Adults	Environment	Meso / Macro	Characteristics Of Living Area	2.03	1.73	1.45	2.18
degree of urbanization	Adults	Environment	Meso / Macro	Characteristics Of Living Area	1.91	1.60	1.33	2.13
area deprivation	Adults	Environment	Meso / Macro	Characteristics Of Living Area	1.77	2.00	1.00	1.80
EM region of origin	Adults	Environment	Meso / Macro	Characteristics Of Living Area	1.69	2.00	1.22	1.22
size of municipality	Adults	Environment	Meso / Macro	Characteristics Of Living Area	1.65	1.73	1.25	1.33
neighbourhood healthy food availability	Adults	Environment	Meso / Macro	Environment Food Availability And Accessibility	2.61	2.45	1.82	2.64
food store access	Adults	Environment	Meso / Macro	Environment Food Availability And Accessibility	2.20	2.45	1.36	2.09
supermarket access	Adults	Environment	Meso / Macro	Environment Food Availability And Accessibility	2.18	2.17	1.58	2.00
shop accessibility	Adults	Environment	Meso / Macro	Environment Food Availability And Accessibility	2.12	2.00	1.57	2.00
small food store availability	Adults	Environment	Meso / Macro	Environment Food Availability And Accessibility	2.04	2.17	1.42	1.83
geographical distance to supermarket	Adults	Environment	Meso / Macro	Environment Food Availability And Accessibility	1.97	1.83	1.33	2.08
EM accessibility to traditional foods	Adults	Environment	Meso / Macro	Environment Food Availability And Accessibility	1.92	2.09	1.18	1.91
shop design	Adults	Environment	Meso / Macro	Environment Food Availability And Accessibility	1.92	2.00	1.23	1.92
spatial distance food-consumer	Adults	Environment	Meso / Macro	Environment Food Availability And Accessibility	1.86	1.73	1.18	2.09
distance to coast	Adults	Environment	Meso / Macro	Environment Food Availability And Accessibility	1.49	1.62	1.08	1.23
neighbourhood supermarket density	Adults	Environment	Meso / Macro	Food Outlet Density	2.27	2.27	1.45	2.36
healthy food store density	Adults	Environment	Meso / Macro	Food Outlet Density	2.01	1.83	1.20	2.40
supermarket density	Adults	Environment	Meso / Macro	Food Outlet Density	1.92	1.85	1.38	1.85
fast food outlet density	Adults	Environment	Meso / Macro	Food Outlet Density	1.90	2.00	1.08	2.08
food outlet density around workplace	Adults	Environment	Meso / Macro	Food Outlet Density	1.81	2.08	1.15	1.62
food outlet density	Adults	Environment	Meso / Macro	Food Outlet Density	1.78	1.92	1.00	1.92
food outlet density around home	Adults	Environment	Meso / Macro	Food Outlet Density	1.73	1.80	1.00	1.90
restaurant density	Adults	Environment	Meso / Macro	Food Outlet Density	1.67	1.83	1.17	1.42
presence of food cues	Adults	Environment	Meso / Macro	Exposure To Food Promotion	2.42	2.42	1.58	2.45
food adverts	Adults	Environment	Meso / Macro	Exposure To Food Promotion	2.28	2.33	1.33	2.50
exposure to food adverts	Adults	Environment	Meso / Macro	Exposure To Food Promotion	2.28	2.36	1.40	2.36
purchase prompts at food outlet	Adults	Environment	Meso / Macro	Exposure To Food Promotion	2.05	1.91	1.55	1.91
market prices	Adults	Environment	Meso / Macro	Market Prices	2.38	1.82	1.55	3.00
cost of healthier market basket	Adults	Environment	Meso / Macro	Market Prices	2.21	2.07	1.43	2.43
food-related NGO activity	Adults	Environment	Meso / Macro	Societal Initiatives	1.78	1.92	1.17	1.67
community-supported agriculture programs	Adults	Environment	Meso / Macro	Societal Initiatives	1.75	2.00	1.07	1.64
nutritional composition guidelines	Adults	Policy	Industry	Industry Regulations	2.35	2.27	1.55	2.45
portion size regulations	Adults	Policy	Industry	Industry Regulations	2.28	1.77	1.69	2.54
food nutritional composition regulations	Adults	Policy	Industry	Industry Regulations	2.11	2.17	1.33	2.17
lobbying	Adults	Policy	Industry	Industry Influence	2.02	1.92	1.36	2.08
market regulations	Adults	Policy	Government	Governmental Regulations	2.26	2.25	1.25	2.67
food advertisement regulations	Adults	Policy	Government	Governmental Regulations	2.24	2.27	1.45	2.27
front-of-pack label regulations	Adults	Policy	Government	Governmental Regulations	2.19	2.42	1.33	2.17
taxes on unhealthy food	Adults	Policy	Government	Governmental Regulations	2.15	1.92	1.45	2.36
subsidies for healthy foods	Adults	Policy	Government	Governmental Regulations	2.13	2.08	1.58	1.92

Determinant	Age Group	Main level	Stem-category	Leaf-category	OPR	Mod	RS	PLE
dietary guidelines	Adults	Policy	Government	Governmental Regulations	2.11	2.57	1.21	1.93
nutrition labeling regulations	Adults	Policy	Government	Governmental Regulations	2.00	2.08	1.27	2.00
food advertisement bans	Adults	Policy	Government	Governmental Regulations	1.96	1.92	1.25	2.08
food label regulations	Adults	Policy	Government	Governmental Regulations	1.92	2.18	1.18	1.82
programs discouraging unhealthy eating	Adults	Policy	Government	Campaigns	2.22	2.08	1.67	2.08
programs promoting healthy eating	Adults	Policy	Government	Campaigns	2.19	2.15	1.62	2.00
educational campaigns for healthy food	Adults	Policy	Government	Campaigns	2.06	2.38	1.25	1.92
sustainability awareness	Adults	Policy	Government	Broader Governmental Policies	N.A.	N.A.	N.A.	N.A.
health awareness	Adults	Policy	Government	Broader Governmental Policies	2.15	2.10	1.50	2.10
EM immigrant-related policy	Adults	Policy	Government	Broader Governmental Policies	1.94	2.18	1.09	2.00

Determinant	Age Group	Main level	Stem-category	Leaf-category	OPR	Mod	RS	PLE
genetic nutrient intolerances	Children	Individual	Biological	Food-Related Physiology	N.A.	N.A.	N.A.	N.A.
hormones	SA children	Individual	Biological	Food-Related Physiology	N.A.	N.A.	N.A.	N.A.
appetite	Children	Individual	Biological	Food-Related Physiology	2.12	1.44	1.88	2.11
food allergies	Children	Individual	Biological	Food-Related Physiology	1.84	1.44	1.75	1.44
taste sensitivity	Children	Individual	Biological	Food-Related Physiology	1.74	1.14	1.67	1.57
obesity-associated genes	Children	Individual	Biological	Food-Related Physiology	1.69	1.00	1.75	1.44
gut microbiota	Children	Individual	Biological	Food-Related Physiology	1.56	1.43	1.17	1.50
BMI	Children	Individual	Biological	Anthropometrics	1.81	1.50	1.29	2.00
body composition	Children	Individual	Biological	Anthropometrics	1.65	1.56	1.38	1.33
birth weight	Children	Individual	Biological	Anthropometrics	1.37	1.11	1.25	1.11
food preferences	Children	Individual	Biological	Sensory Perception	2.44	2.22	1.63	2.67
taste aversions	Children	Individual	Biological	Sensory Perception	2.14	1.50	1.86	2.13
food liking	Children	Individual	Biological	Sensory Perception	2.13	1.89	1.75	1.89
food wanting	Children	Individual	Biological	Sensory Perception	2.12	1.63	1.57	2.38
fat liking	Children	Individual	Biological	Sensory Perception	2.07	1.75	1.71	1.88
learned taste preferences	Children	Individual	Biological	Sensory Perception	2.05	1.25	1.86	2.13
biological taste preferences	Children	Individual	Biological	Sensory Perception	1.81	1.33	1.63	1.67
mental health status	Children	Individual	Biological	Physical Health	N.A.	N.A.	N.A.	N.A.
health status	Children	Individual	Biological	Physical Health	1.92	1.56	1.63	1.78
chronic diseases	Children	Individual	Biological	Physical Health	1.84	1.11	1.75	1.78
prematurity	Children	Individual	Biological	Physical Health	1.37	1.00	1.25	1.22
sleep duration	Children	Individual	Biological	Sleep Characteristics	1.57	1.89	1.00	1.33
sleep difficulties	Children	Individual	Biological	Sleep Characteristics	1.53	1.78	1.13	1.11
having been breastfed	Children	Individual	Demographic	Biological Demographics	1.88	1.25	1.43	2.25
sex	Children	Individual	Demographic	Biological Demographics	1.61	1.00	1.38	1.78
age	Children	Individual	Demographic	Biological Demographics	1.59	1.00	1.25	1.89
cultural identity	SA children	Individual	Demographic	Cultural Characteristics	1.90	1.14	1.43	2.43
religion	Children	Individual	Demographic	Cultural Characteristics	1.71	1.00	1.43	2.00
nationality	Children	Individual	Demographic	Cultural Characteristics	1.67	1.00	1.43	1.88
ethnicity	Children	Individual	Demographic	Cultural Characteristics	1.63	1.00	1.43	1.75
EM competency in host language	Children	Individual	Demographic	Situational Demographics	1.69	2.00	1.17	1.33
place of residence	Children	Individual	Demographic	Situational Demographics	1.58	1.11	1.38	1.56
socio-economic status	Children	Individual	Demographic	Personal Socio-Economic Status	1.82	1.00	1.50	2.22
self-efficacy	Children	Individual	Psychological	Personality	2.12	2.00	1.50	2.11
distractibility when eating	Children	Individual	Psychological	Personality	1.96	2.00	1.50	1.63
self-esteem	SA children	Individual	Psychological	Personality	1.60	1.33	1.13	1.78
personality / temperament	Children	Individual	Psychological	Personality	1.59	1.25	1.43	1.38
wellbeing	Children	Individual	Psychological	Mood And Emotions	N.A.	N.A.	N.A.	N.A.
positive emotions	Children	Individual	Psychological	Mood And Emotions	1.89	1.88	1.29	1.88
negative emotions	Children	Individual	Psychological	Mood And Emotions	1.76	1.63	1.43	1.50
mood	Children	Individual	Psychological	Mood And Emotions	1.70	1.63	1.14	1.75
depressive symptomatology	SA children	Individual	Psychological	Mood And Emotions	1.62	1.43	1.43	1.29
self-regulation skills	Children	Individual	Psychological	Self-Regulation	2.24	1.78	1.88	2.13
self-control	Children	Individual	Psychological	Self-Regulation	1.98	1.89	1.38	2.00
emotion suppression	Children	Individual	Psychological	Self-Regulation	1.79	1.86	1.29	1.57
impulsivity	Children	Individual	Psychological	Self-Regulation	1.75	1.43	1.50	1.57
future time orientation	SA children	Individual	Psychological	Self-Regulation	1.72	1.67	1.00	2.00
executive functioning	Children	Individual	Psychological	Self-Regulation	1.53	1.17	1.17	1.67
previous experience with disease	Children	Individual	Psychological	Health Cognitions	N.A.	N.A.	N.A.	N.A.
nutrition knowledge	SA children	Individual	Psychological	Food Knowledge, Skills and Abilities	2.13	2.56	1.38	1.78
food familiarity	Children	Individual	Psychological	Food Knowledge, Skills and Abilities	2.03	2.11	1.25	2.11
food memories	Children	Individual	Psychological	Food Knowledge, Skills and Abilities	1.60	1.25	1.29	1.63
food involvement	Children	Individual	Psychological	Food Beliefs	2.23	2.38	1.71	1.75
food concerns	SA children	Individual	Psychological	Food Beliefs	2.16	2.00	1.57	2.13
food enjoyment	Children	Individual	Psychological	Food Beliefs	2.02	2.11	1.38	1.89
trust in the food industry	SA children	Individual	Psychological	Food Beliefs	1.93	1.88	1.29	2.00
food risk aversion	SA children	Individual	Psychological	Food Beliefs	1.81	1.88	1.29	1.63
habitual eating	Children	Individual	Psychological	Food Habits	2.03	1.89	1.63	1.78
past eating behavior	Children	Individual	Psychological	Food Habits	1.80	1.27	1.60	1.73
mindful eating	SA children	Individual	Psychological	Eating Regulation	2.01	2.25	1.43	1.63
external eating	Children	Individual	Psychological	Eating Regulation	1.87	1.75	1.57	1.50
neophobia	Children	Individual	Psychological	Eating Regulation	1.86	1.50	1.71	1.50

Determinant	Age Group	Main level	Stem-category	Leaf-category	OPR	Mod	RS	PLE
food selectivity	Children	Individual	Psychological	Eating Regulation	1.77	1.86	1.17	1.71
emotional eating	Children	Individual	Psychological	Eating Regulation	1.77	1.25	1.71	1.50
variety seeking	Children	Individual	Psychological	Eating Regulation	1.71	1.86	1.14	1.57
intuitive eating	Children	Individual	Psychological	Eating Regulation	1.70	1.63	1.14	1.75
body weight perception	SA children	Individual	Psychological	Weight Control Cognitions And Behaviors	N.A.	N.A.	N.A.	N.A.
eating in the absence of hunger	Children	Individual	Psychological	Weight Control Cognitions And Behaviors	2.24	2.00	2.00	1.71
weight control goal	SA children	Individual	Psychological	Weight Control Cognitions And Behaviors	1.95	1.78	1.38	2.00
body dissatisfaction	SA children	Individual	Psychological	Weight Control Cognitions And Behaviors	1.83	1.50	1.75	1.38
weight control concerns	SA children	Individual	Psychological	Weight Control Cognitions And Behaviors	1.77	1.75	1.29	1.63
cognitive restraint	SA children	Individual	Psychological	Weight Control Cognitions And Behaviors	1.74	1.57	1.29	1.71
disinhibition	Children	Individual	Psychological	Weight Control Cognitions And Behaviors	1.71	1.71	1.33	1.43
satiation	Children	Individual	Situational	Hunger	N.A.	N.A.	N.A.	N.A.
food deprivation	Children	Individual	Situational	Hunger	2.22	1.88	1.86	2.00
hunger	Children	Individual	Situational	Hunger	2.21	1.38	2.00	2.25
frequency of television viewing	Children	Individual	Situational	Related Health Behaviors	2.34	2.22	1.50	2.56
physical activity level	Children	Individual	Situational	Related Health Behaviors	2.22	2.33	1.63	1.89
perceived stress	SA children	Individual	Situational	Situational And Time Constraints	2.24	2.25	1.57	2.13
daily rhythm/structure	Children	Individual	Situational	Situational And Time Constraints	1.86	2.00	1.14	1.88
parental access to a car	Children	Individual	Situational	Situational And Time Constraints	1.42	1.38	1.00	1.38
household size	Children	Interpersonal	Social	Family Structure	1.56	1.00	1.29	1.75
family cohesion	Children	Interpersonal	Social	Family Structure	1.49	1.22	1.13	1.56
family composition	Children	Interpersonal	Social	Family Structure	1.23	1.00	1.13	1.00
family food culture	Children	Interpersonal	Social	Family Food Culture	2.07	1.67	1.63	2.11
household food processing (cooking)	Children	Interpersonal	Social	Family Food Culture	2.05	2.11	1.50	1.78
family preferences	Children	Interpersonal	Social	Family Food Culture	2.01	1.78	1.50	2.00
household food production (growing)	Children	Interpersonal	Social	Family Food Culture	1.42	1.50	1.00	1.25
parental food insecurity	Children	Interpersonal	Social	Household Socio-Economic Status	1.93	2.25	1.29	1.63
household food security	Children	Interpersonal	Social	Household Socio-Economic Status	1.91	1.50	1.57	1.88
low parental income	Children	Interpersonal	Social	Household Socio-Economic Status	1.79	1.00	1.50	2.11
parental educational level	Children	Interpersonal	Social	Household Socio-Economic Status	1.72	1.11	1.38	2.00
household income	Children	Interpersonal	Social	Household Socio-Economic Status	1.72	1.00	1.38	2.11
household socio-economic status	Children	Interpersonal	Social	Household Socio-Economic Status	1.70	1.00	1.25	2.22
household budget constraints	Children	Interpersonal	Social	Household Socio-Economic Status	1.67	1.25	1.43	1.63
parental income	Children	Interpersonal	Social	Household Socio-Economic Status	1.65	1.00	1.38	1.89
parental occupation	Children	Interpersonal	Social	Household Socio-Economic Status	1.45	1.00	1.13	1.67
peer modeling	Children	Interpersonal	Social	Social Influence	2.28	2.00	1.75	2.22
eating occasion	Children	Interpersonal	Social	Social Influence	2.21	2.13	1.43	2.38
social norms	Children	Interpersonal	Social	Social Influence	2.05	1.33	1.50	2.56
decisional power in groups	SA children	Interpersonal	Social	Social Influence	1.85	1.78	1.25	1.89
parental recommendations	Children	Interpersonal	Social	Social Support	2.37	2.38	1.57	2.38
social support	Children	Interpersonal	Social	Social Support	2.06	2.00	1.38	2.11
community recommendations	Children	Interpersonal	Social	Social Support	1.93	1.88	1.29	2.00
peer cooking practices and skills	SA children	Interpersonal	Social	Social Support	1.86	2.25	1.14	1.63
salesperson recommendations	SA children	Interpersonal	Social	Social Support	1.74	2.00	1.14	1.50
bonding with parents	Infants	Interpersonal	Social	Social Support	1.70	1.50	1.14	1.88
parental nutrition knowledge	Children	Interpersonal	Social	Parental Resources And Risk Factors	2.37	2.56	1.63	2.11
parental food market knowledge	Children	Interpersonal	Social	Parental Resources And Risk Factors	2.24	2.56	1.38	2.11
parental food product knowledge	Children	Interpersonal	Social	Parental Resources And Risk Factors	2.19	2.33	1.50	2.00
parental time constraints	Children	Interpersonal	Social	Parental Resources And Risk Factors	1.85	1.56	1.25	2.11
parental depression	Children	Interpersonal	Social	Parental Resources And Risk Factors	1.29	1.25	1.00	1.13
parental weight control concerns	Children	Interpersonal	Social	Parental Attitudes And Beliefs	2.12	2.00	1.57	2.00
parental perception of child's weight	Children	Interpersonal	Social	Parental Attitudes And Beliefs	2.06	2.44	1.38	1.67
parental perceived food safety	Children	Interpersonal	Social	Parental Attitudes And Beliefs	2.02	2.33	1.38	1.67
parental weight control goal	Children	Interpersonal	Social	Parental Attitudes And Beliefs	1.96	2.00	1.43	1.75
parental lay food theories	Children	Interpersonal	Social	Parental Attitudes And Beliefs	1.94	1.71	1.50	1.86
parental trust in food labeling	Children	Interpersonal	Social	Parental Attitudes And Beliefs	1.78	2.00	1.14	1.63
parental trust in food producers	Children	Interpersonal	Social	Parental Attitudes And Beliefs	1.78	2.00	1.14	1.63
parental food ethics	Children	Interpersonal	Social	Parental Attitudes And Beliefs	1.71	1.25	1.43	1.75
parental food risk aversion	Children	Interpersonal	Social	Parental Attitudes And Beliefs	1.70	1.88	1.14	1.50
parental willingness-to-pay	Children	Interpersonal	Social	Parental Attitudes And Beliefs	1.70	1.63	1.17	1.71
parental trust in food certification	Children	Interpersonal	Social	Parental Attitudes And Beliefs	1.61	1.75	1.14	1.38
parental trust in food distribution	Children	Interpersonal	Social	Parental Attitudes And Beliefs	1.55	1.86	1.00	1.29

Determinant	Age Group	Main level	Stem-category	Leaf-category	OPR	Mod	RS	PLE
parental body dissatisfaction	Children	Interpersonal	Social	Parental Attitudes And Beliefs	1.49	1.50	1.14	1.25
parental modeling	Children	Interpersonal	Social	Parental Behaviors	2.40	2.25	1.71	2.38
parental food habits	Children	Interpersonal	Social	Parental Behaviors	2.33	2.11	1.63	2.44
parental food processing (cooking)	Children	Interpersonal	Social	Parental Behaviors	2.28	2.22	1.75	2.00
EM parental conformity to tradition	Children	Interpersonal	Social	Parental Behaviors	2.00	1.33	1.67	2.17
parental lifestyle	Children	Interpersonal	Social	Parental Behaviors	2.00	1.67	1.63	1.89
parental smart shopping	Children	Interpersonal	Social	Parental Behaviors	1.92	2.00	1.25	1.89
parental frugality	Children	Interpersonal	Social	Parental Behaviors	1.64	1.29	1.50	1.38
parental food production (growing)	Children	Interpersonal	Social	Parental Behaviors	1.51	1.44	1.25	1.22
early exposure	Children	Interpersonal	Social	Parental Feeding Styles	N.A.	N.A.	N.A.	N.A.
parental portion size habits	Children	Interpersonal	Social	Parental Feeding Styles	2.27	2.22	1.50	2.33
food used as incentive	Children	Interpersonal	Social	Parental Feeding Styles	2.24	2.38	1.57	2.00
parental food restriction	Children	Interpersonal	Social	Parental Feeding Styles	2.18	2.22	1.63	1.89
parental pressure-to-eat	Children	Interpersonal	Social	Parental Feeding Styles	2.15	2.22	1.63	1.78
parental instrumental feeding	Children	Interpersonal	Social	Parental Feeding Styles	2.11	1.88	1.71	1.88
parental complementary feeding practices	Infants	Interpersonal	Social	Parental Feeding Styles	2.04	2.00	1.57	1.75
parental emotional feeding	Children	Interpersonal	Social	Parental Feeding Styles	1.95	1.63	1.57	1.88
cultural beliefs	Children	Interpersonal	Cultural	Cultural Cognitions	2.14	1.14	1.71	2.71
cultural values	Children	Interpersonal	Cultural	Cultural Cognitions	2.10	1.14	1.71	2.57
cultural norms	Children	Interpersonal	Cultural	Cultural Cognitions	1.93	1.14	1.57	2.29
social role of food	SA children	Interpersonal	Cultural	Cultural Cognitions	1.90	1.43	1.43	2.14
cultural food customs	Children	Interpersonal	Cultural	Cultural Behaviors	2.08	1.33	1.50	2.67
cultural traditions	Children	Interpersonal	Cultural	Cultural Behaviors	1.98	1.14	1.57	2.43
religious rituals	Children	Interpersonal	Cultural	Cultural Behaviors	1.71	1.00	1.43	2.00
EM level of acculturation	SA children	Interpersonal	Cultural	Cultural Behaviors	1.56	1.33	1.00	1.83
nutritional composition	SA children	Environment	Product	Intrinsic Product Attributes	2.26	2.14	1.67	2.14
product taste	Children	Environment	Product	Intrinsic Product Attributes	2.24	2.25	1.57	2.13
product texture	Children	Environment	Product	Intrinsic Product Attributes	2.16	2.00	1.57	2.13
product sensory properties	Children	Environment	Product	Intrinsic Product Attributes	2.10	2.14	1.33	2.14
product flavor	Children	Environment	Product	Intrinsic Product Attributes	2.02	2.13	1.29	2.00
product package size	Children	Environment	Product	Extrinsic Product Attributes	2.49	2.63	1.57	2.50
package size	Children	Environment	Product	Extrinsic Product Attributes	2.42	2.63	1.43	2.50
price	SA children	Environment	Product	Extrinsic Product Attributes	2.40	2.00	1.71	2.63
nutritional information	SA children	Environment	Product	Extrinsic Product Attributes	2.23	2.56	1.50	1.89
product appearance	Children	Environment	Product	Extrinsic Product Attributes	2.23	2.38	1.29	2.38
package color	Children	Environment	Product	Extrinsic Product Attributes	2.20	2.88	1.14	2.00
product variety	Children	Environment	Product	Extrinsic Product Attributes	2.17	2.25	1.43	2.13
product packaging	Children	Environment	Product	Extrinsic Product Attributes	2.11	2.38	1.14	2.25
product convenience	SA children	Environment	Product	Extrinsic Product Attributes	2.02	2.00	1.38	2.00
food labeling	Children	Environment	Product	Extrinsic Product Attributes	2.01	2.33	1.13	2.00
product brand	SA children	Environment	Product	Extrinsic Product Attributes	1.89	2.00	1.29	1.75
portion size	Children	Environment	Micro	Portion Size	2.41	2.22	1.63	2.56
food accessibility	Children	Environment	Micro	Home Food Availability And Accessibility	2.64	2.44	1.88	2.67
food availability	Children	Environment	Micro	Home Food Availability And Accessibility	2.64	2.33	1.88	2.78
product visibility	SA children	Environment	Micro	Home Food Availability And Accessibility	2.07	2.22	1.25	2.11
meal environment	Children	Environment	Micro	Eating Environment	2.21	2.44	1.38	2.11
time of day	Children	Environment	Meso / Macro	Natural Conditions	1.67	1.25	1.43	1.63
season	Children	Environment	Meso / Macro	Natural Conditions	1.54	1.00	1.38	1.56
weather	Children	Environment	Meso / Macro	Natural Conditions	1.28	1.00	1.00	1.33
area deprivation	Children	Environment	Meso / Macro	Characteristics Of Living Area	1.64	1.29	1.29	1.71
EM region of origin	Children	Environment	Meso / Macro	Characteristics Of Living Area	1.61	1.00	1.33	1.83
degree of urbanization	Children	Environment	Meso / Macro	Characteristics Of Living Area	1.49	1.00	1.14	1.75
school canteen food environment	SA children	Environment	Meso / Macro	Environment Food Availability And Accessibility	2.24	2.14	1.33	2.57
shop accessibility	SA children	Environment	Meso / Macro	Environment Food Availability And Accessibility	2.06	1.56	1.38	2.56
shop design	SA children	Environment	Meso / Macro	Environment Food Availability And Accessibility	1.90	2.00	1.13	2.00
fast food outlet density	SA children	Environment	Meso / Macro	Food Outlet Density	1.91	1.33	1.38	2.33
supermarket density	SA children	Environment	Meso / Macro	Food Outlet Density	1.78	1.44	1.13	2.22
exposure to food adverts	SA children	Environment	Meso / Macro	Exposure To Food Promotion	2.27	2.00	1.50	2.56
purchase prompts at food outlet	SA children	Environment	Meso / Macro	Exposure To Food Promotion	2.08	1.89	1.50	2.11
market prices	SA children	Environment	Meso / Macro	Market Prices	2.12	1.56	1.50	2.56
food-related NGO activity	Children	Environment	Meso / Macro	Societal Initiatives	1.67	1.75	1.00	1.75
portion size regulations	Children	Policy	Industry	Industry Regulations	2.36	1.88	1.71	2.63

Determinant	Age Group	Main level	Stem-category	Leaf-category	OPR	Mod	RS	PLE
food nutritional composition regulations	Children	Policy	Industry	Industry Regulations	2.18	2.13	1.29	2.50
nutritional composition guidelines	Children	Policy	Industry	Industry Regulations	1.99	2.38	1.14	1.88
lobbying	Children	Policy	Industry	Industry Influence	2.04	1.86	1.17	2.50
dietary guidelines	SA children	Policy	Government	Governmental Regulations	2.23	2.22	1.50	2.22
food advertisement regulations	Children	Policy	Government	Governmental Regulations	2.17	2.00	1.38	2.44
subsidies for healthy foods	Children	Policy	Government	Governmental Regulations	2.17	2.11	1.38	2.33
nutrition labeling regulations	Children	Policy	Government	Governmental Regulations	2.01	2.11	1.13	2.22
market regulations	Children	Policy	Government	Governmental Regulations	1.99	1.63	1.14	2.63
food label regulations	Children	Policy	Government	Governmental Regulations	1.95	2.13	1.14	2.00
food advertisement bans	Children	Policy	Government	Governmental Regulations	1.88	1.56	1.25	2.22
programs promoting healthy eating	Children	Policy	Government	Campaigns	2.28	2.78	1.38	2.00
programs discouraging unhealthy eating	Children	Policy	Government	Campaigns	2.14	2.56	1.25	2.00
educational campaigns for healthy food	Children	Policy	Government	Campaigns	2.11	2.33	1.25	2.11
